# Hyperspectral inversion of winter wheat nitrogen content based on feature optimization and stacking ensemble learning

**DOI:** 10.3389/fpls.2026.1911340

**Published:** 2026-09-01

**Authors:** Fuxing Guan, Changchun Li, Guijun Yang, Yinghua Jiao, Guangsheng Zhang, Dan Li, Tengyue Cui

**Affiliations:** 1 School of Surveying and Land Information Engineering, Henan Polytechnic University, Jiaozuo, China; 2 College of Geological Engineering and Geomatics, Chang’an University, Xi’an, China; 3 Shandong Provincial Institute of Land Surveying and Mapping, Jinan, China; 4 College of Surveying and Mapping Engineering, Heilongjiang Institute of Technology, Harbin, Heilongjiang, China

**Keywords:** ensemble learning, feature selection, hyperspectral remote sensing, machine learning, nitrogen, SHAP analysis, winter wheat

## Abstract

Rapid, non-destructive crop nitrogen monitoring is a core pillar of precision fertilization and mass-balance nutrient management, which requires real-time uptake data to balance fertilizer inputs, crop demand and environmental nitrogen losses. But hyperspectral data redundancy, background noise and black-box machine learning models limit inversion generalization performance and interpretability, hampering field-scale mass-balance management. Using jointing-to-filling winter wheat canopy hyperspectral data, we built a three-tier progressive framework of preprocessing, feature selection and model architecture, systematically evaluating 2 preprocessing, 5 feature selection and 5 modeling schemes, with SHAP-based analysis to reveal the physiological mechanisms underlying band contributions. The main findings are summarized as follows: (1) First-derivative (FD) transformation achieved selective enhancement of nitrogen signals. By removing indirect coupling confounding signals, the test-set *R*² of all models increased from 0.57–0.73 under Savitzky–Golay (SG) full-spectrum input to 0.71–0.76. (2) Feature selection delivered a significantly greater accuracy gain than model optimization, and a clear feature-model compatibility pattern was identified. Specifically, the sparse high-purity features selected by LASSO were highly aligned with the similarity measurement mechanism of the GPR kernel function. (3) The FD–LASSO–stacking ensemble scheme achieved the optimal inversion performance, with a test-set *R*² of 0.875, RMSE of 0.281%, and RPD of 2.834. This model also achieved favorable performance with an R^2^ of 0.781 in cross-growth-stage inversion, which verifies its generalization ability and stability to a certain extent.(4) SHAP analysis quantified the contribution levels of features screened by each algorithm, and identified cross-algorithm common core bands including the 757 nm red edge and 939 nm near-infrared bands, verifying the biological rationality of model decisions. This study not only realizes high-accuracy nitrogen inversion in winter wheat, but also deepens the mechanistic understanding of hyperspectral inversion from the perspectives of feature-model compatibility and model interpretability. It provides theoretical and technical support for lightweight sensor band design, field precision nitrogen diagnosis, and mass-balance-based nitrogen management.

## 1 Introduction

Winter wheat is one of the most dominant staple food crops in China, and its stable, high-yield production holds strategic importance for safeguarding national food security and advancing sustainable agricultural development. Nitrogen (N) is the primary limiting factor regulating photosynthetic production, canopy establishment, and grain yield and quality formation in wheat, and mass-balance-based nutrient management has emerged as the core framework for reconciling wheat productivity with environmental protection in intensive cropping systems. Real-time, accurate, and non-destructive monitoring of crop N status serves as a critical technical prerequisite for implementing variable-rate fertilization, improving nitrogen use efficiency (NUE), reducing agricultural non-point source pollution, and operationalizing field-scale mass-balance N management. Owing to its capability to continuously characterize the fine spectral responses of crop canopies and leaves across the visible (Vis), near-infrared (NIR), and short-wave infrared (SWIR) regions, hyperspectral remote sensing has emerged as an essential tool for non-destructive diagnosis of crop N status. Wan et al. (2022) achieved cross-species leaf nitrogen concentration (LNC) estimation by integrating transfer learning with hyperspectral reflectance analysis, demonstrating the cross-scene generalization potential of hyperspectral information in N inversion. Liu et al. (2023) further synchronously retrieved multiple leaf nutrient elements in crops using airborne hyperspectral data across multiple years, validating the reliability of hyperspectral data for synergistic monitoring of multiple crop nutrients. Nevertheless, the collaborative optimization of each stage in the data processing pipeline and model interpretability remain key bottlenecks restricting the engineering application of hyperspectral N inversion.

Regarding spectral preprocessing, methods such as Savitzky–Golay (SG) smoothing, First Derivative (FD) transformation, and fractional-order derivative transformation are widely employed to suppress instrument noise, correct baseline drift, and enhance weak absorption features. Sun et al. (2021) proposed fractional-order SG derivatives combined with wavelength selection to estimate leaf water content in maize, confirming that fine adjustment of derivative orders can significantly improve the correlation between spectra and biochemical parameters. Yang et al. (2025b) systematically compared different fractional-order derivative preprocessing methods for chlorophyll density estimation in winter wheat, showing that derivative transformation can enhance the correlation between spectra and biochemical parameters to varying degrees and optimize modeling accuracy. Tuerxun et al. (2023) combined derivative preprocessing with dimensionality reduction algorithms for chlorophyll content retrieval in jujube leaves, further corroborating the effect of derivative-domain feature enhancement on improving inversion accuracy. Zhang et al., 2021b) introduced a transfer learning framework into hyperspectral estimation of winter wheat chlorophyll content, indirectly reflecting the critical importance of preprocessing and feature representation for cross-condition modeling robustness. However, existing studies predominantly validate preprocessing effects from the perspective of accuracy improvement, while systematic elucidation of the intrinsic regulatory mechanism by which FD transformation selectively enhances direct N physiological responses and decouples indirect confounding signals from water and dry matter in the derivative domain remains lacking. This gap makes it difficult to theoretically explain the root causes of accuracy differences under different preprocessing methods from a signal perspective.

In terms of feature selection, algorithms including competitive adaptive reweighted sampling (CARS), genetic algorithm (GA), least absolute shrinkage and selection operator (LASSO), variable importance in projection (VIP), and uninformative variable elimination (UVE) are extensively applied to extract sensitive wavelengths, thereby reducing model complexity and mitigating collinearity and overfitting. Tian et al. (2022) constructed a canopy hyperspectral feature selection and fusion framework for winter wheat and soil total N, verifying the efficacy of feature optimization in improving N diagnosis accuracy. Yang et al. (2025a) proposed a novel feature selection algorithm integrating genetic algorithms, exponential decay functions, and machine learning to achieve high-precision inversion of winter wheat leaf area index, demonstrating the advantages of search-based algorithms in high-dimensional wavelength selection. Cui et al. (2025) systematically investigated the coupling effect of spectral resolution and multiple feature selection methods on equivalent water thickness estimation in fruit tree leaves, revealing the regulatory relationship between feature subset structure and model performance. Li et al. (2025a) proposed a multi-level feature selection strategy for cotton in arid regions, which significantly improved N content estimation accuracy. Similarly, Xiao et al. (2025) combined feature selection with machine learning for cotton N nutrition index estimation, validating the dual benefits of dimensionality reduction for both model efficiency and accuracy. Wang et al. (2021) achieved cotton leaf N content estimation by combining wavelengths sensitive to N concentration and oxidase activity, while Azadnia et al. (2023) employed wavelength optimization coupled with machine learning for rapid estimation of N, phosphorus, and potassium contents in apple tree leaves. Collectively, these studies demonstrate that physiology-oriented wavelength selection represents an effective approach for improving inversion accuracy and developing lightweight sensors. Nevertheless, most existing studies validate selection performance under a single model, and systematic comparisons of feature structural differences across multiple mainstream algorithms within a unified experimental framework remain scarce. In particular, quantitative analysis of the adaptation law between “feature subset structural characteristics” and “model inductive bias” is lacking. How structural differences in feature subsets generated by different selection algorithms—including wavelength number, spectral interval distribution, and redundancy level—affect the inversion performance of various model types remains to be systematically elucidated.

For model construction, machine learning methods such as random forest (RF), support vector regression (SVR), extreme gradient boosting (XGBoost), and Gaussian process regression (GPR) have gradually replaced traditional partial least squares regression (PLSR). Stacking ensemble learning further improves inversion accuracy and robustness by leveraging the error complementarity of heterogeneous base learners. Lin and Liu (2022) proposed an improved random forest method based on hybrid weight learning, which significantly enhanced the accuracy of hyperspectral soil total N estimation. Wang et al. (2022a) inverted winter wheat chlorophyll content using ground-measured hyperspectral data combined with multiple machine learning algorithms, while Song et al. (2024) achieved N content estimation by mining spatial-spectral features of maize leaf veins. Both studies confirmed the advantages of machine learning in characterizing nonlinear hyperspectral relationships. Chen et al. (2025) further integrated near-ground hyperspectral and meteorological information to construct a winter wheat plant N concentration prediction model. Zhao et al. (2025) introduced a spatial attention mechanism into one-dimensional convolutional neural networks for UAV-based hyperspectral inversion of winter wheat LNC, and Du et al. (2023) proposed an incremental learning framework for crop growth parameter estimation and N diagnosis, reflecting recent advances in deep and incremental learning for N inversion. Longmire et al. (2022) fused airborne hyperspectral and thermal infrared data to retrieve crop traits for predicting wheat grain protein, while Zhang et al. (2025a) combined machine learning to estimate wheat N nutrition index. Regarding ensemble learning, Deng et al. (2025) constructed a stacking ensemble framework integrating multisource data for sorghum yield prediction in arid regions, and Kong et al. (2026) combined multi-source feature selection with stacking ensemble learning for maize N monitoring. Both studies confirmed that ensemble models exhibit higher accuracy and robustness than single models when processing high-dimensional heterogeneous data. However, current ensemble learning research predominantly focuses on optimizing model structure itself, with few studies conducting whole-pipeline collaborative optimization integrating preprocessing strategies, multi-algorithm feature selection, and ensemble modeling. The contribution magnitude and synergistic mechanisms of each pipeline stage remain unclear. More critically, machine learning-based inversion generally suffers from the “black box” problem, making it difficult to interpret the physiological basis of model decisions and wavelength contribution mechanisms, which limits the credibility and engineering application of inversion results.

Interpretable analysis provides a new technical pathway to address this challenge. The SHAP (SHapley Additive exPlanations) method, based on game theory, can quantify the marginal contribution of each input feature to model outputs, transforming complex black-box models into interpretable decision tools. Kong et al. (2026) utilized SHAP in maize N monitoring to reveal that red-edge and NIR wavelengths are the core contributors to N prediction, with analytical results highly consistent with crop N physiological mechanisms. Deng et al. (2025) employed SHAP in sorghum yield prediction to quantify the differential contributions of spectral features across growth stages. Peng et al. (2026) combined data augmentation with interpretable machine learning for leaf protein N estimation in rice, further validating the value of SHAP in identifying key driving wavelengths and enhancing model transparency. Additionally, Flynn et al (Flynn et al., 2023), Oswald et al. (2024), and Zhang et al. (2021a) explored quantitative associations between hyperspectral features and crop physicochemical parameters in legume biomass and N accumulation, apricot canopy N radiative transfer modeling, and winter wheat leaf water inversion, respectively, providing collateral evidence for mechanistic interpretation of N inversion. Overall, SHAP interpretability analysis has been applied in hyperspectral inversion for maize, sorghum, rice, and other crops, but systematic research in winter wheat LNC inversion remains limited. Existing studies mostly use SHAP as an auxiliary presentation of results, rather than validating feature selection mechanisms and delineating physiological response windows from the perspective of feature contribution. The association between statistically optimal feature selection and crop N physiological response mechanisms remains to be established.

**Figure 1:**
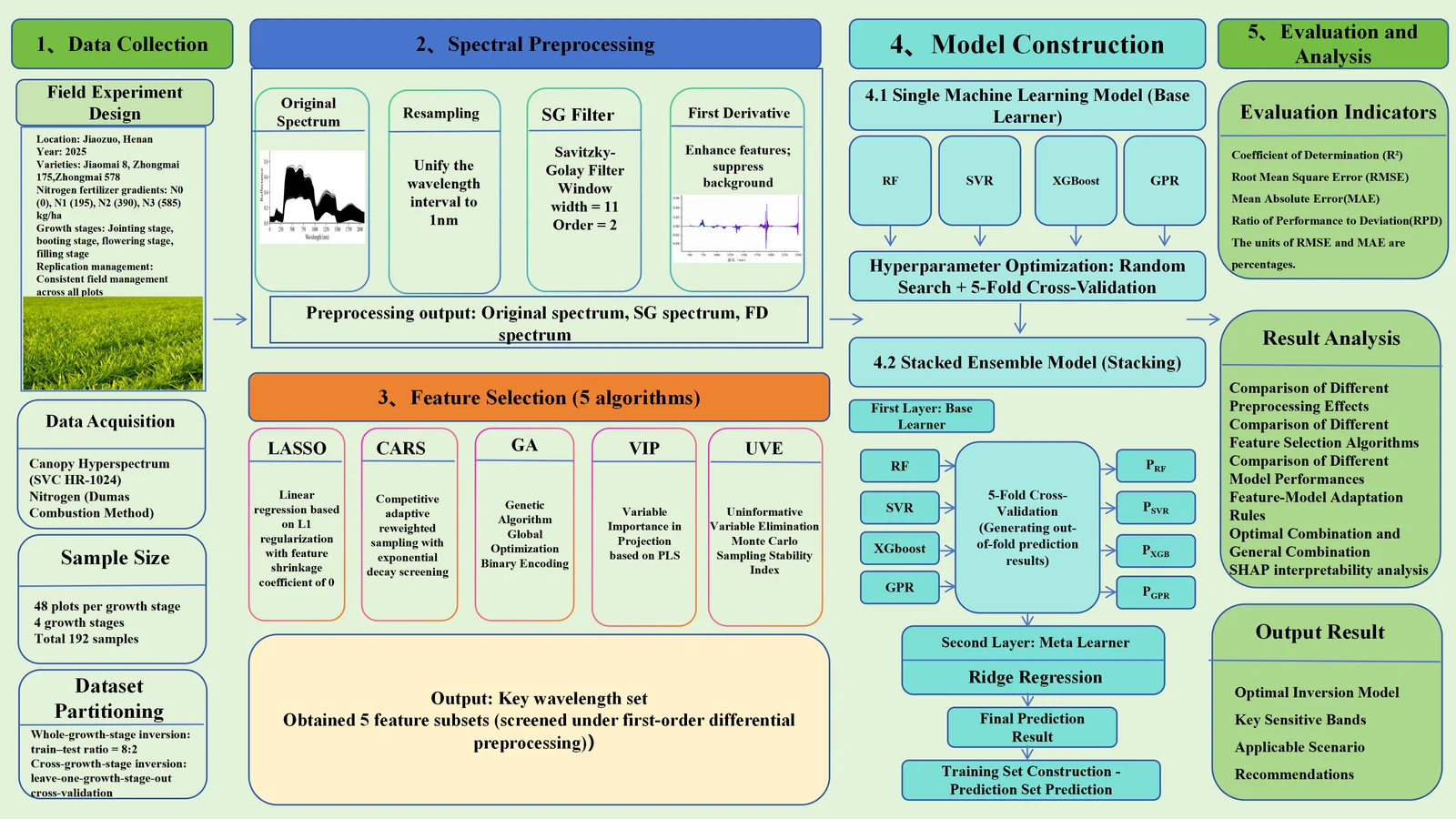
Overall technical roadmap of the study.

To address the aforementioned gaps, this study was conducted based on winter wheat field experiments in Jiaozuo City, Henan Province, using winter wheat leaves from the jointing to grain-filling stages as research objects. A three-tier progressive inversion framework integrating spectral preprocessing, feature selection, and model architecture was constructed. The specific objectives were to: (1) compare the variation patterns of spectral-N correlation under SG smoothing and FD transformation preprocessing, and analyze the regulatory effect of FD transformation on N spectral signals; (2) systematically compare the wavelength selection results of five feature selection algorithms (CARS, GA, LASSO, VIP, UVE) within a unified framework, and quantify the adaptation relationship between feature structure and model performance by combining inversion accuracies of four single models (RF, SVR, XGBoost, GPR) and the Stacking ensemble model; (3) introduce SHAP interpretability analysis to quantify the contribution hierarchy of core wavelengths under each feature subset, and explain model decision mechanisms from the perspective of physiological responses. This study aims to identify a high-accuracy and interpretable winter wheat LNC inversion scheme, providing a reference for hyperspectral precision monitoring of winter wheat N, wavelength selection for lightweight sensor development, and robust technical support for field-scale mass-balance nitrogen management. The technical roadmap of this study is presented in **Figure 1**.

## 2 Materials and methods

### 2.1 Experimental design

**Figure 2:**
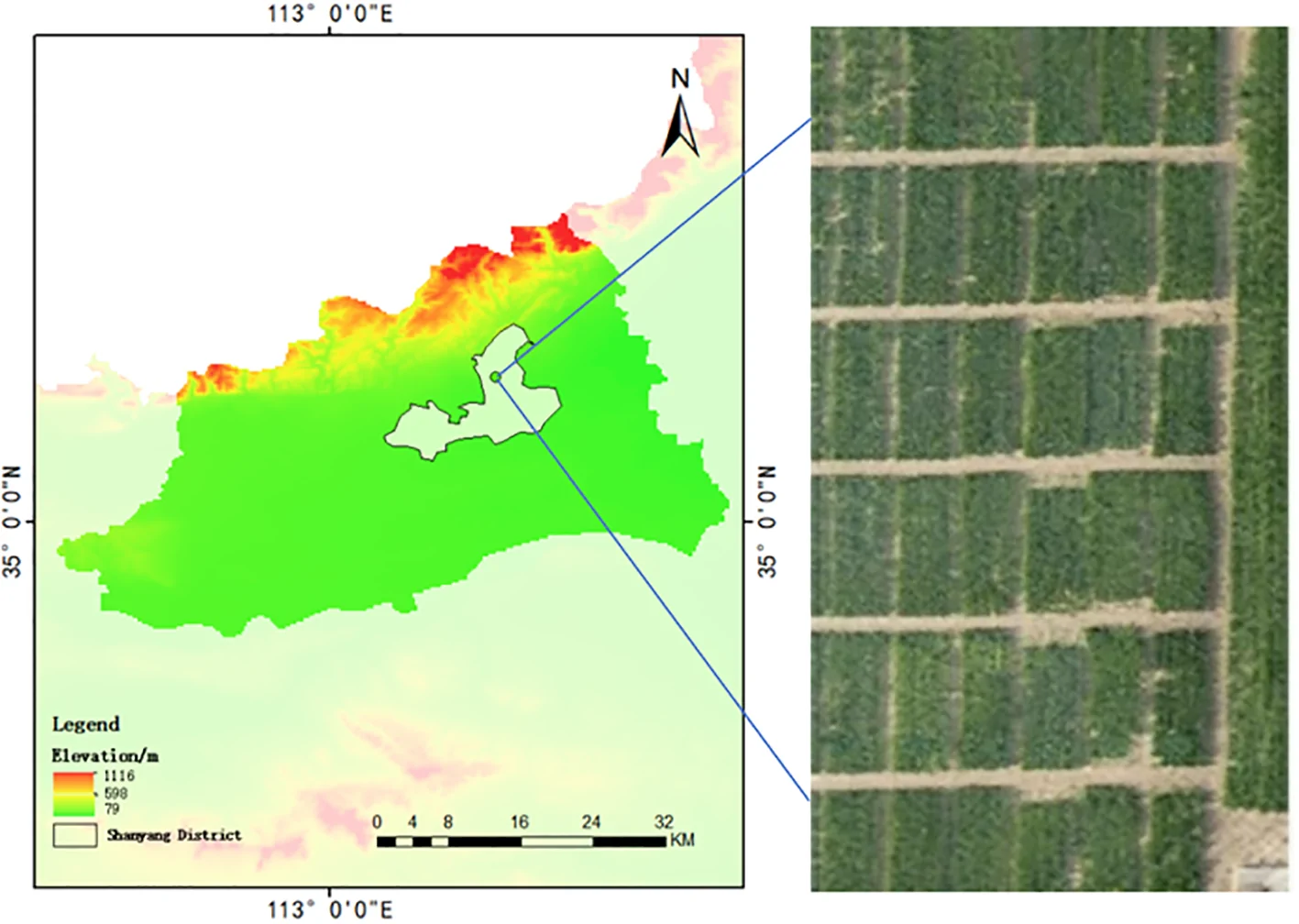
Schematic diagram of winter wheat study area in Jiaozuo City, Henan Province.

This study was carried out in 2025 at the Precision Agriculture Demonstration Base of Jiaozuo Academy of Agricultural and Forestry Sciences, Jiaozuo City, Henan Province, China. The study site is shown in **Figure 2**, located at 35°8′23″ N, 113°15′4″ E. Three winter wheat cultivars were included in the experiment: Jiaomai 8, Zhongmai 175, and Zhongmai 578. The trial comprised 48 experimental plots (1.4 m × 9.5 m each), with a uniformly controlled wheat sowing density of approximately 260 plants m^−^². Buffer strips were set between adjacent plots to eliminate mutual interference. The nitrogen application gradient was designed in accordance with the Henan Provincial Local Standard Technical Specification for Nitrogen Loss Mitigation in Wheat-Maize Rotation Farmlands of Northern Henan (DB41/T 2862-2025). For the dominant winter wheat yield range of 6750–9000 kg·ha^−^¹ in northern Henan, this standard recommends a nitrogen application rate of 165–210 kg N·ha^−^¹. In this study, 195 kg N·ha^−^¹ — the median value of the recommended interval and a rate widely applied in local agricultural production — was adopted as the conventional nitrogen level. Four nitrogen supply treatments were set as follows: N0 (0 kg N·ha^−^¹, zero-nitrogen treatment to create severe nitrogen deficiency stress), N1 (195 kg N·ha^−^¹, the locally recommended conventional nitrogen application rate), N2 (390 kg N·ha^−^¹, approximately twice the conventional rate to simulate high nitrogen input scenarios), and N3 (585 kg N·ha^−^¹, approximately three times the conventional rate to establish excessive nitrogen supply conditions).With the exception of the nitrogen gradient treatments, all plots received the same basal fertilization: 375 kg ha^−^¹ calcium superphosphate and 150 kg ha^−^¹ potassium sulfate (Zhang et al., 2022). Both basal and nitrogen fertilizers were applied in a single dose, with no surface mulching practiced throughout the crop growing season, and field management practices were kept consistent across all plots.

### 2.2 Data collection

In 2025, canopy hyperspectral reflectance data acquired by the SVC spectrometer and ground-measured nitrogen content data were collected simultaneously at four key growth stages of winter wheat: jointing stage (March 21), booting stage (April 16), flowering stage (April 28), and grain-filling stage (May 7). Measurements from all 48 experimental plots were treated as independent samples at each stage, yielding a total of 192 samples for the entire experiment.

#### 2.2.1 Hyperspectral data acquisition

Canopy spectral measurements were collected using an SVC HR-1024i portable field spectroradiometer (Spectra Vista Corporation, Longmont, CO, USA), which covers a spectral detection range of 350–2500 nm (Herr and Carter, 2023). A 25°field of view (FOV) was adopted for this study, configured via the FIBER1 external fiber-optic probe channel. The 25°FOV of the standard fiber probe is a widely accepted specification in field hyperspectral remote sensing practice. All spectral data were acquired between 10:00 and 14:00 local time on clear, cloudless days to ensure stable incident solar radiation. During measurements, the probe was held vertically downward at a height of 0.5 m above the crop canopy. White reference calibration was performed with a manufacturer-supplied Spectralon diffuse reflectance reference panel (sintered polytetrafluoroethylene, PTFE) every 15 minutes, or immediately following any noticeable change in weather conditions. Raw spectral data were processed using the dedicated SVC software package to derive canopy hyperspectral reflectance. For each experimental plot, three sampling points were randomly selected. At each point, spectral data were recorded for 5 seconds and automatically averaged into a single spectral curve by the instrument software. The final reflectance value for each plot was calculated as the mean reflectance across the three sampling points.

#### 2.2.2 Leaf nitrogen sampling

For leaf nitrogen content determination of winter wheat, destructive plant sampling was first conducted in each experimental plot. The harvested samples were immediately transported to the laboratory and placed in a forced-air drying oven. Enzyme inactivation was performed at 105 °C for 30 min, after which samples were dried to constant weight at 80 °C. The aboveground biomass of the dried samples was then weighed. Following biomass measurement, the dried samples were ground into a fine, homogeneous powder for subsequent nitrogen analysis. Nitrogen content was quantified via the Dumas combustion method using a Dumas nitrogen analyzer (Thermo Fisher Scientific, Inc., Longmont, CO, USA) (Berger et al., 2020). The instrument determines sample nitrogen content through a sequential workflow of high-temperature combustion, reduction, gas separation, and detection. The calculation formula is shown in Equation 1:

(1) $$N(\%)=\frac{m}{M}*100\%$$

Where *N* (%) denotes the nitrogen content of the sample; *m* denotes the mass of nitrogen measured by the analyzer; and *M* denotes the mass of the dried test sample.

### 2.3 Data preprocessing

To minimize the confounding effects of instrument noise, soil background, and atmospheric scattering, the raw hyperspectral data were preprocessed through resampling, SG smoothing, and FD transformation. All preprocessing workflows were executed in MATLAB R2023b.

#### 2.3.1 Savitzky-Golay filtering

SG filter is a smoothing technique grounded in local polynomial least-squares fitting. It retains critical spectral features including peaks and edge profiles while suppressing noise, and is widely adopted for hyperspectral data preprocessing (Savitzky and Golay, 1964).

In this study, the SG filter was configured with a window size of 11 and a polynomial order of 2. Its core principle is to fit *N* data points within a sliding window using a polynomial of order *p*, as shown in Equation 2:

(2) $$f(x)=a_{0}+a_{1}x+a_{2}x^{2}+\ldots +a_{p}x^{p}$$

Where *x* denotes the local coordinate within the sliding window. The coefficients *a*_0_, *a*_1_,…, *a*_p_ are estimated via the least squares method, and the fitted value at the window center is taken as the final smoothed output.

#### 2.3.2 Derivative transformation

FD transformation is a core technique for hyperspectral feature enhancement. It quantifies the rate of change in reflectance spectra to uncover subtle spectral information embedded in raw data, effectively mitigating extraneous background noise and amplifying nitrogen-sensitive spectral features (Song et al., 2023). The calculation formula is shown in Equation 3:

(3) $$R^{\prime }(\lambda_{i})=\frac{R(\lambda_{i+1})-R(\lambda_{i-1})}{\lambda_{i+1}-\lambda_{i-1}}$$

Where $R(\lambda_{i+1})$ denotes the reflectance at wavelength $\lambda_{i+1}$; and $R(\lambda_{i-1})$ denotes the reflectance at wavelength $\lambda_{i-1}$.

### 2.4 Feature selection

Feature selection aims to eliminate redundant noise from massive hyperspectral datasets and screen out feature bands strongly correlated with winter wheat nitrogen content. It not only improves model inversion accuracy and computational efficiency, but also serves as a widely adopted approach for mitigating overfitting and enhancing model stability and interpretability.

#### 2.4.1 Least absolute shrinkage and selection operator

LASSO is a linear regression technique grounded in L1 regularization. By introducing an L1-norm penalty on feature coefficients into the loss function, it shrinks the coefficients of irrelevant features to zero, making it well suited for feature selection in high-dimensional hyperspectral data (Asad et al., 2026). Its optimization objective function is shown in Equation 4:

(4) $$\underset{\beta_{0},\beta }{\min}\sum_{i=1}^{n}{\left(y_{i}-\beta_{0}-\sum_{j=1}^{p}x_{ij}\beta_{j}\right)}^{2}+\lambda \sum_{j=1}^{p}\left|\beta_{j}\right|$$

Where *n* denotes the number of samples; *p* denotes the number of features; *y_i_* denotes the observed value of the *i*-th sample; *x_ij_* denotes the value of the *i*-th sample for the *j*-th feature; *β*_0_ denotes the intercept term; *β_j_* denotes the regression coefficient of the *j*-th feature; *β* denotes the coefficient vector; *λ* denotes the regularization parameter. The term $\sum_{i=1}^{n}{\left(y_{i}-\beta_{0}-\sum_{j=1}^{p}x_{ij}\beta_{j}\right)}^{2}$ is the residual sum of squares loss term, and $\sum_{j=1}^{p}|\beta_{j}|$ is the L1 regularization term.

#### 2.4.2 Variable importance in projection

VIP is a feature selection technique grounded in PLSR. It screens informative features by quantifying each feature’s contribution to the predictive performance of the PLSR model. A higher VIP value indicates a more significant contribution of the corresponding feature to the model’s explained variance and prediction accuracy (Huang et al., 2023). The VIP value of the j-th feature is defined as shown in Equation 5:

(5) $$VIP_{j}=\sqrt{\frac{p\sum_{t-1}^{k}(R_{t}^{2}\cdot w_{jt}^{2})}{\sum_{t-1}^{k}R_{t}^{2}}}$$

Where *p* denotes the total number of hyperspectral features; *t* denotes the number of latent variables; *k* denotes the index of latent variables; $R_{t}^{2}$ denotes the proportion of response variance explained by the *t*-th latent component; and $w_{jt}^{2}$ denotes the weight coefficient of the *j*-th feature on the *t*-th latent component.

#### 2.4.3 Uninformative variable elimination

UVE is a variable selection algorithm grounded in PLSR. By introducing random noise variables to construct an extended dataset, it calculates a stability index for the regression coefficient of each variable during model training, and eliminates original variables with low stability using the stability distribution of noise variables as the discrimination criterion. The core rationale of UVE is to identify informative features that contribute significantly to the model by maximizing the stability of variable regression coefficients, thereby improving model robustness and predictive performance (Wang et al., 2022b).

#### 2.4.4 Competitive adaptive reweighted sampling

CARS is a classic variable selection algorithm for high-dimensional data. It combines Monte Carlo sampling with PLSR to quantify variable importance based on regression coefficients, and progressively eliminates redundant and noisy variables via adaptive reweighting and exponential decay strategies. Using root mean squared error of cross-validation (RMSECV) as the evaluation metric, the algorithm determines the optimal feature subset, which can effectively reduce data dimensionality and mitigate variable collinearity (Hu et al., 2025).

#### 2.4.5 Genetic algorithm

GA is an intelligent optimization algorithm inspired by the principles of biological evolution. By simulating core genetic operations including selection, crossover, and mutation, it performs iterative optimization in the feature space with a predefined objective function as the evaluation criterion, progressively screening out the optimal feature subset. Possessing robust global search capability, GA can effectively handle high-dimensional, non-linear data with complex collinearity. It reduces feature dimensionality while retaining valid information from raw data (Thorp et al., 2017).

### 2.5 Model construction

Four representative machine learning models, namely RF, SVR, XGBoost, and GPR, were selected as base learners to construct a stacking ensemble model. The inversion performances of individual models and the ensemble model were compared and analyzed. Model hyperparameters were optimized via random search combined with cross-validation. The full dataset was split into training and test subsets at an 8:2 ratio. All modeling procedures were implemented in Python 3.9.

#### 2.5.1 Single machine learning models

1. RF is an ensemble tree model based on the bagging framework. It constructs multiple decision trees through bootstrap sampling, and derives the final prediction by averaging the outputs of all individual trees. This mechanism effectively reduces model variance and improves generalization performance (Wei et al., 2025).
2. SVR is built on the principle of structural risk minimization. It maps high-dimensional spectral features into a Hilbert space via the kernel trick, and constructs a regression function in the high-dimensional feature space that satisfies the structural risk minimization criterion (Soltanikazemi et al., 2022).
3. XGBoost is a gradient-boosted tree model grounded in the boosting framework. It iteratively trains weak learners to correct the residuals of preceding models, and incorporates regularization terms to suppress overfitting (Huber et al., 2022).
4. GPR is a non-parametric Bayesian probabilistic model. It directly models the distribution of the target function and provides uncertainty estimates alongside prediction outputs, making it well suited for modeling tasks with small sample sizes and high-dimensional data (Pourreza et al., 2025).

#### 2.5.2 Stacking ensemble model

A two-layer stacking regression ensemble learning framework was employed in this study. The first layer comprises four heterogeneous base learners: RF, SVR, XGBoost, and GPR. The hyperparameters of all base learners were optimized via random search. Meta-features, defined as the individual prediction outputs of each base model, were generated on the training set using 5-fold cross-validation to avoid data leakage. The second layer adopts RidgeCV as the meta-learner. It takes the prediction outputs of the four base models as input features and the observed true values as the response variable to fit a linear fusion relationship. For test set inference, all base learners first generate prediction meta-features from the test data. These meta-features are then fed into the RidgeCV meta-learner to produce the final ensemble prediction. The meta-learner adaptively learns the optimal fusion weight for each base model, thereby leveraging the complementary strengths of individual models (Zhang et al., 2023). The detailed mathematical formulation of the stacking ensemble workflow is as follows:

(6) $$B\;=\{f_{1}(\text{RF}),f_{2}(\text{SVR}),f_{3}(\text{XGBoost}),f_{4}(\text{GPR})\}$$

Equation 6 defines the set of base models, where *B* denotes the collection of all base learners in the first layer; and *f*_1_, *f*_2_, *f*_3_, and *f*_4_ denote the four heterogeneous regression models with hyperparameters optimized via random search, respectively.

(7) $$\phi (x)={\left[f_{1}(x),f_{2}(x),f_{3}(x),f_{4}(x)\right]}^{T}$$

Equation 7 defines the base model prediction outputs for a single sample, where ϕ(x) denotes the meta-feature vector; *x* denotes a single standardized input feature vector; $f_{k}(x)$ denotes the predicted value for sample *x* generated by the *k*-th base model; and the ${\left[\ldots \right]}^{T}$ denotes the vector transpose operation.

(8) $${\hat{y}}_{\text{stack}}(x)=w_{1}f_{1}(x)+w_{2}f_{2}(x)+w_{3}f_{3}(x)+w_{4}f_{4}(x)+b$$

Equation 8 describes the fusion prediction formula of the Ridge regression meta-model, where ${\hat{y}}_{\text{stack}}(x)$ denotes the final predicted value output by the stacking ensemble model; *w*_1_、*w*_2_、*w*_3_ and *w*_4_ denote the fusion weights corresponding to the prediction outputs of the four base learners; and *b* denotes the intercept term fitted by the Ridge regression model.

(9) $$\underset{w,b}{\min}\sum_{i=1}^{N}{\left(y_{i}-\sum_{k=1}^{4}w_{k}f_{k}(x_{i})-b\right)}^{2}+\lambda \sum_{k=1}^{4}w_{k}^{2}$$

Equation 9 formulates the RidgeCV loss function, which serves as the optimization objective for deriving the fusion weights: where *N* denotes the total number of training samples; *y_i_* denotes the observed value of the i-th sample; *b* denotes the intercept term; *λ* denotes the regularization parameter. The term $\sum_{i=1}^{N}{\left(y_{i}-\sum_{k=1}^{4}w_{k}f_{k}(x_{i})-b\right)}^{2}$ is the residual sum of squares (i.e., the prediction error term), and $\sum_{k=1}^{4}w_{k}f_{k}(x_{i})+b$ is the fitted value of the i-th sample generated by the meta-model. The term $\lambda \sum_{k=1}^{4}w_{k}^{2}$ is the L2 regularization penalty term. The $\underset{w,b}{\min}$​ operator minimizes the overall loss function with respect to the fusion weights *w*_1_~*w*_4_ and the intercept *b*, yielding the optimal fusion parameters.

### 2.6 Accuracy evaluation metrics

(1) Coefficient of Determination (*R*²): *R*² quantifies the goodness of fit between model-inversed values and field-measured values. It ranges from 0 to 1, and a higher *R*² indicates better inversion accuracy of the model (Zhu et al., 2025). The specific calculation formula is shown in Equation 10:

(10) $$R^{2}=1-\frac{\sum_{i=1}^{n}{\left(x_{i}-y_{i}\right)}^{2}}{\sum_{i=1}^{n}{\left(x_{i}-\bar{x}\right)}^{2}}$$

(2) Root Mean Squared Error (RMSE): RMSE measures the magnitude of deviation between model-inversed values and measured values. A smaller RMSE corresponds to higher model accuracy (Zhu et al., 2025). In this study, RMSE is expressed in percentage (%). The specific calculation formula is shown in Equation 11:

(11) $$RMSE=\sqrt{\frac{\sum_{i=1}^{n}{\left(x_{i}-y_{i}\right)}^{2}}{n}}$$

(3) Mean Absolute Error (MAE): MAE is defined as the average of absolute differences between measured values and model-inversed values. A smaller MAE indicates a lower overall inversion error and better predictive performance of the model (Zhu et al., 2025). In this study, MAE is expressed in percentage (%). The specific calculation formula is shown in Equation 12:

(12) $$MAE=\frac{1}{n}\sum_{i=1}^{n}\left|x_{i}-y_{i}\right|$$

(4) Residual Prediction Deviation (RPD): RPD is calculated as the ratio of the standard deviation of measured sample values to the standard error of model predictions, as shown in Equations 13–15 (Zhu et al., 2025).

(13) $$SD=\sqrt{\frac{1}{n-1}\sum_{i=1}^{n}{\left(x_{i}-\bar{x}\right)}^{2}}$$

(14) $$SEP=\sqrt{\frac{1}{n}\sum_{i=1}^{n}{\left(x_{i}-y_{i}\right)}^{2}}$$

(15) $$RPD=\frac{SD}{SEP}$$

Where *x_i_* denotes the measured nitrogen content of the *i*-th winter wheat sample; *y_i_* denotes the predicted nitrogen content of the *i*-th winter wheat sample; $\bar{x}$ denotes the mean of measured winter wheat nitrogen content; *n* denotes the total number of samples; *SD* denotes the standard deviation of measured values; and *SEP* denotes the standard error of prediction.

### 2.7 Correlation analysis

The Pearson correlation coefficient (commonly denoted as *r*) is a statistical metric that quantifies the direction and strength of linear correlation between two continuous variables. It is widely applied in agricultural remote sensing to screen spectral bands sensitive to crop biochemical components (e.g., winter wheat nitrogen content) (Zhang et al., 2025b).For *n* paired observations (*x_i_*, *y_i_*), the Pearson correlation coefficient is calculated as shown in Equation 16:

(16) $$r=\frac{\sum_{i=1}^{n}(x_{i}-\bar{x})(y_{i}-\bar{y})}{\sqrt{\sum_{i=1}^{n}{\left(x_{i}-\bar{x}\right)}^{2}}\sqrt{\sum_{i=1}^{n}{\left(y_{i}-\bar{y}\right)}^{2}}}$$

Where *x_i_* and *y_i_* denote the observed values of the two variables for the *i*-th sample, respectively; $\bar{x}$ and $\bar{y}$ denote the corresponding sample means.

### 2.8 Interpretability analysis

SHAP is a classic local model interpretability method grounded in the Shapley value from cooperative game theory. Following the additivity principle, it decomposes the prediction of an individual sample into a linear sum of a global baseline value and the SHAP contribution of each input feature (Dong et al., 2025).By calculating the marginal contribution of each feature across all possible feature subsets and weighting contributions by the permutation probability of each subset, SHAP quantifies the positive or negative driving strength of individual features on sample-level predictions. This mechanism enables rigorous feature attribution analysis for black-box machine learning models. The core mathematical formulation is defined as shown in Equation 17:

(17) $$\phi_{\text{i}}=\sum_{S\subseteq F\backslash \{i\}}\frac{|S|!\left(|F|-|S|-1\right)!}{|F|!}\left[v\left(S\cup \{i\}\right)-v(S)\right]$$

Where $\phi_{i}\;$ denotes the SHAP value of the *i*-th feature (i.e., its contribution to model prediction); |*F*| denotes the total number of input features; |*S*| denotes the size of feature subset *S*; *S* denotes an arbitrary feature subset excluding the *i*-th feature; and *v*(*S*) denotes the expected prediction value of the model fitted with feature subset *S*. The factorial term $\frac{|S|!(|F|-|S|-1)!}{|F|!}$ is the weighting coefficient, and [v(S∪{i})−v(S)] represents the marginal contribution term.

## 3 Results and analysis

### 3.1 Statistical characteristics of dataset and spectral response analysis

#### 3.1.1 Analysis of nitrogen content in winter wheat

**Figure 3:**
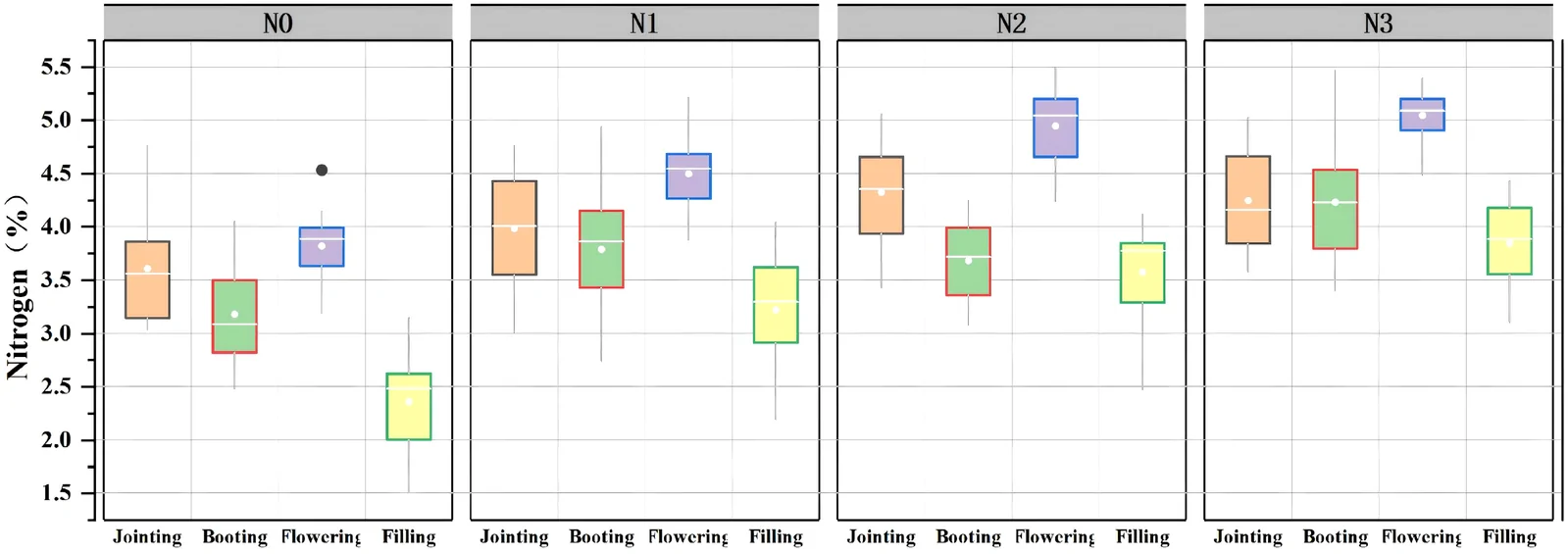
Boxplots of nitrogen content.

**Figure 4:**
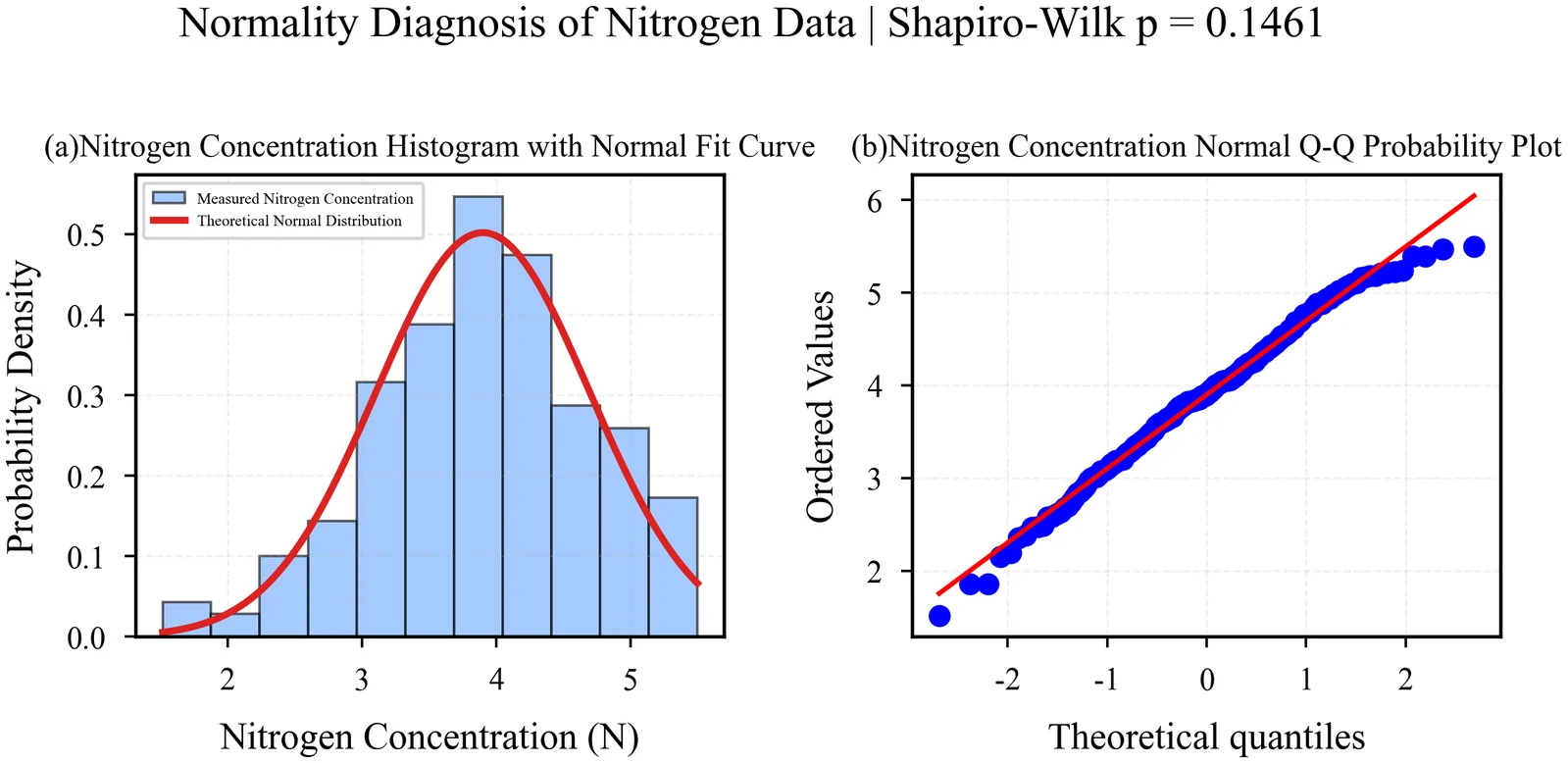
Normality assessment of nitrogen concentration data. **(a)** Histogram of measured nitrogen concentration with fitted theoretical normal_distribution curve; **(b)** Normal Q_Q probability plot for nitrogen concentration.

**Figure 3** shows boxplots of leaf nitrogen content, with clear gradients across nitrogen treatments and no obvious skewness in the full dataset. Mean and median values were closely matched across all treatment and growth stage combinations, confirming a symmetric distribution; median lines fell centrally within the interquartile range (IQR) for most groups. Data dispersion declined markedly with rising nitrogen rates. Medium and high nitrogen treatments (N2, N3) showed compact distributions, with anthesis-stage IQR of only 0.2–0.4%, indicating low variability. A single high-value outlier was found at anthesis in the N0 treatment, with negligible impact on the overall distribution. **Figure 4** presents normality validation of leaf nitrogen content via the Shapiro–Wilk test, probability density histograms, and normal Q–Q plots. The Shapiro–Wilk test returned a p-value of 0.1461 (above the 0.05 significance threshold), so the null hypothesis of normal distribution cannot be rejected. The probability density histogram matched the theoretical normal curve well, forming a standard bell shape with no apparent skewness. In the Q–Q plot, most sample points clustered along the reference diagonal, with only minor deviations at extreme values and no systematic departure from normality.

Overall, the nitrogen dataset has a stable distribution and well-controlled outliers, providing a reliable basis for subsequent inversion modeling and statistical analysis.

#### 3.1.2 Ground hyperspectral data and correlation analysis

**Figure 5:**
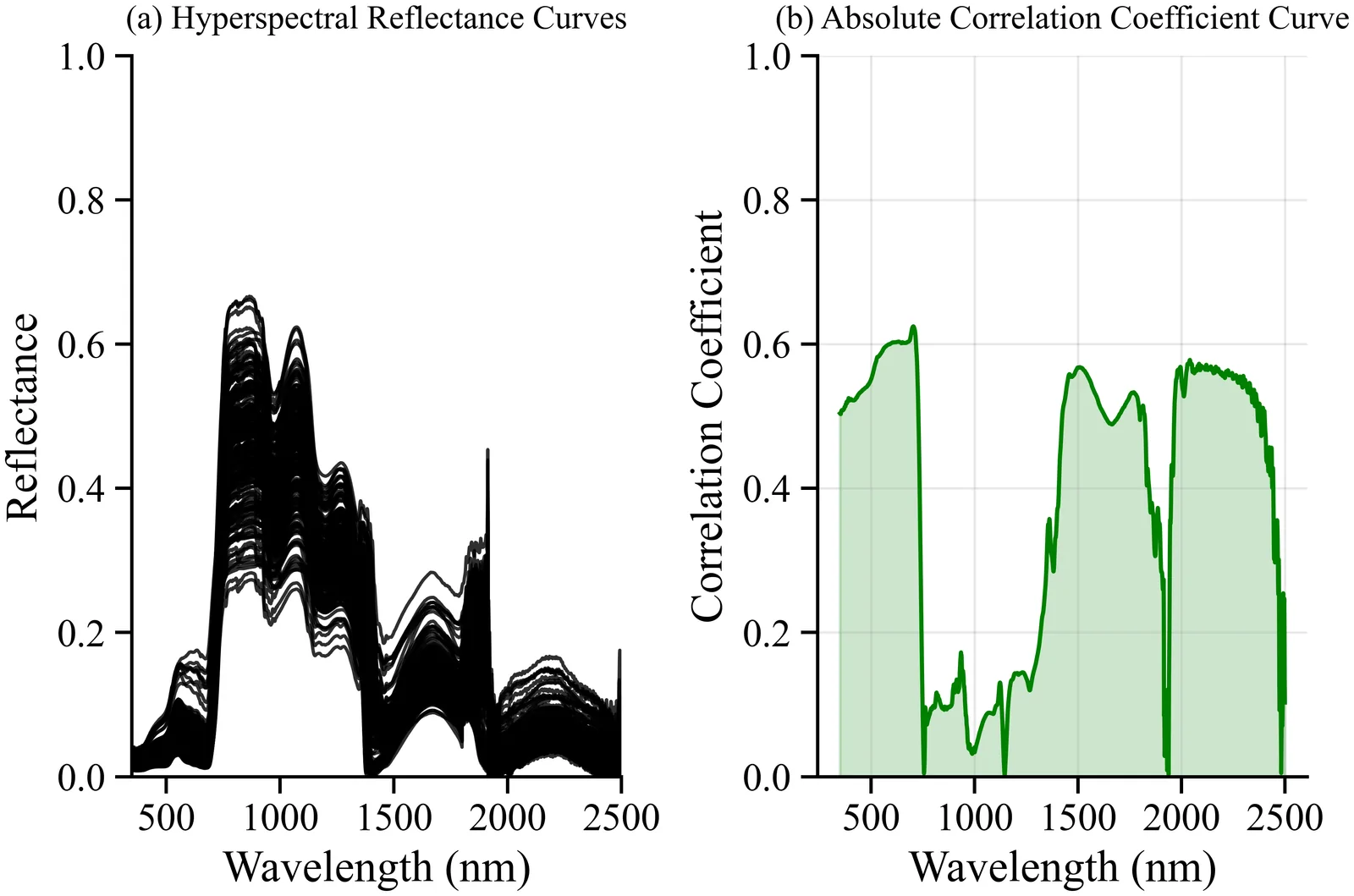
**(a)** SG-filtered spectral curves of winter wheat; **(b)** Pearson correlation plots of spectra and nitrogen.

Hyperspectral data of winter wheat leaves across the full spectral range (350–2500 nm) were preprocessed via resampling combined with SG filtering (**Figure 5a**). This preprocessing pipeline effectively suppresses random instrumental noise and smooths spectral profiles, while fully preserving fine critical spectral features: the red edge inflection point at 680–750 nm, the near-infrared reflectance plateau at 750–900 nm, and the strong water absorption troughs at 1450 nm and 1940 nm. It provides a high-quality data foundation for analyzing the correlation between spectral reflectance and leaf nitrogen concentration. Based on the preprocessed raw reflectance spectra, leaf nitrogen concentration and spectral reflectance exhibited a distinct band-specific differentiation pattern characterized by three high-correlation and three low-correlation regions. The full-spectrum correlation curve was smooth and continuous (**Figure 5b**). The first broad high-correlation plateau occurred in the visible–red edge–early near-infrared region (350–900 nm), with correlation coefficients generally above 0.5. The peak correlation appeared in the red edge region near 700 nm, with a maximum linear correlation coefficient of approximately 0.62 across the full spectrum. The first low-correlation trough was located at 900–1100 nm, where correlation coefficients dropped rapidly to below 0.1. A second high-correlation plateau formed in the early shortwave infrared region (1100–1800 nm), with a peak value near 1500–1600 nm approaching 0.6. The second low-correlation trough occurred at 1800–1950 nm, with correlation coefficients falling back to below 0.2. A third high-correlation plateau was observed in the late shortwave infrared region (1950–2400 nm), with a peak near 2100–2200 nm also approaching 0.6. Beyond 2400 nm, correlation declined rapidly with increasing wavelength. Nevertheless, raw spectra in the sensitive 500–900 nm interval are susceptible to low-frequency interferences including soil background, canopy specular reflection, and baseline drift. These interferences dilute the authentic spectral response driven by nitrogen-regulated changes in chlorophyll content. Meanwhile, the high correlations observed in the shortwave infrared bands (1100–1800 nm and 1950–2400 nm) do not originate solely from the direct response to nitrogen. Instead, they are largely attributed to indirect physiological coupling between nitrogen and leaf water, cellulose, and biomass, introducing prominent confounding signal interference.

**Figure 6:**
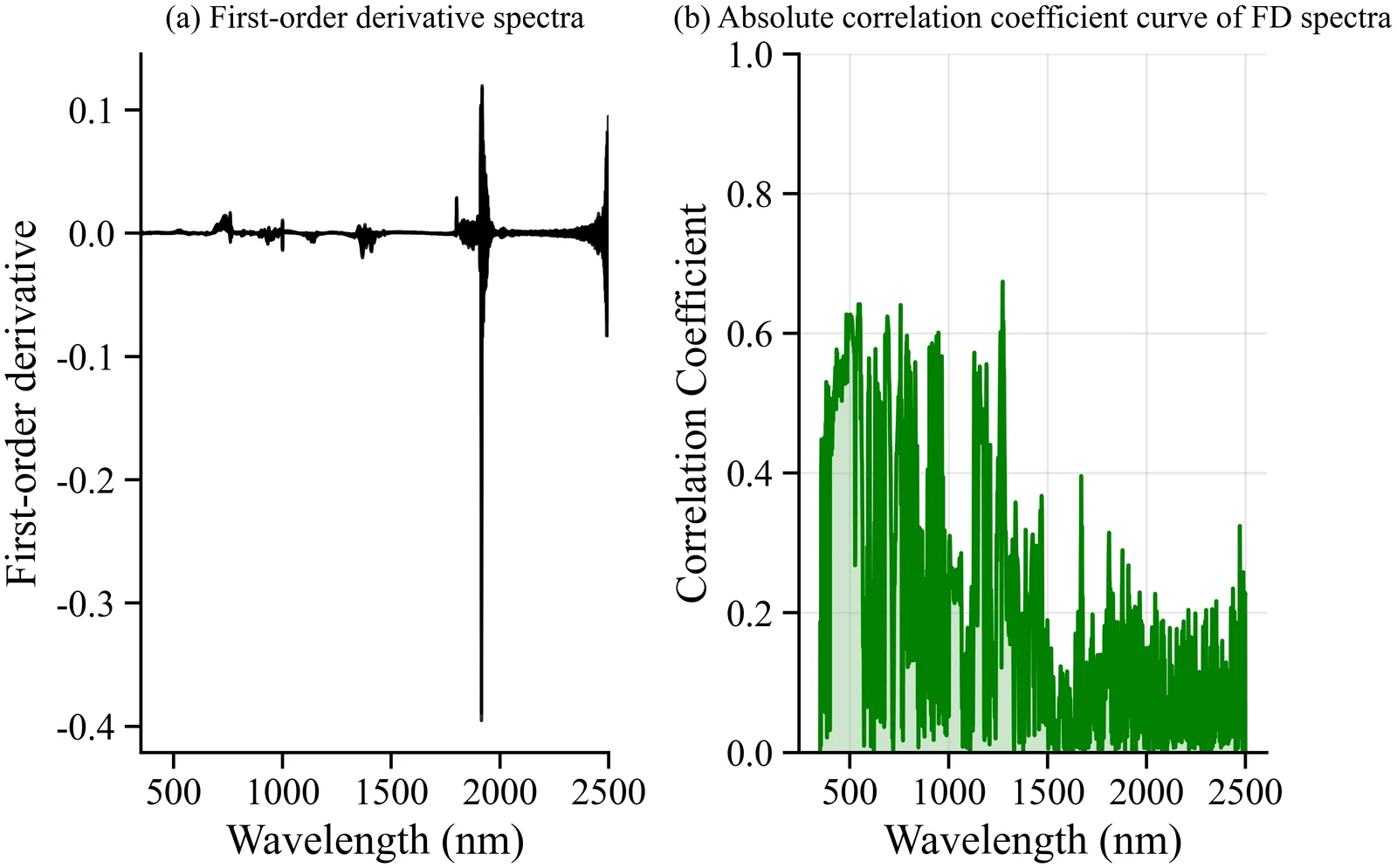
**(a)** FD-processed spectral curves of winter wheat; **(b)** Pearson correlation plots of spectra and nitrogen.

FD spectral transformation was further applied to the SG-preprocessed hyperspectral data. This transformation effectively eliminated baseline drift and low-frequency background interference across the full 350–2500 nm spectral range, amplified subtle variations in spectral slope across all wavebands, and fundamentally reshaped the correlation response pattern between spectral reflectance and leaf nitrogen concentration at different wavelengths (**Figure 6a**). Compared with raw reflectance spectra, FD transformation markedly restructured the band distribution of full-spectrum correlations (**Figure 6b**). Dense clusters of sharp high-correlation peaks emerged in the visible–red edge–early near-infrared region (400–1100 nm), with absolute correlation coefficients generally above 0.4 and numerous peaks exceeding 0.6. The overall detection sensitivity was significantly improved relative to raw reflectance: FD transformation accurately captured red edge slope shifts and visible-band absorption feature changes induced by nitrogen surplus or deficiency, and effectively removed background noise interference that persisted in this interval of the original spectra. Notably, an isolated sharp peak with the highest correlation across the full spectrum appeared in the distal near-infrared region (1200–1300 nm), with a peak correlation coefficient of 0.65–0.7. This band became the most sensitive feature region for nitrogen response after FD transformation. Conversely, FD transformation substantially weakened nitrogen response signals in the mid-to-late shortwave infrared region. Correlation declined rapidly with increasing wavelength beyond 1300 nm, falling below 0.2 at approximately 1500 nm. The 1500–2500 nm shortwave infrared interval generally exhibited low-correlation fluctuations, with only scattered minor rebound peaks near 1700 nm, 2200 nm, and 2500 nm. The underlying mechanism is twofold. First, the strong water absorption bands at 1450 nm and 1940 nm exhibit greatly amplified slope variations under FD transformation, which completely masks the weak spectral signals of nitrogen. Second, FD transformation eliminates the indirect slow-varying response signals in the shortwave infrared region of raw spectra, which originate from the physiological coupling of nitrogen, water, and dry matter. Only a small number of local sensitive peaks corresponding to the direct absorption of protein N–H and C–N bonds are retained. Collectively, these effects produce a differentiated band response pattern: dense high correlations at 500–1100 nm, a single peak extremum at 1200–1300 nm, and overall attenuation beyond 1300 nm.

### 3.2 Feature selection results

#### 3.2.1 Filtering of highly correlated features based on Pearson correlation coefficient

**Figure 7:**
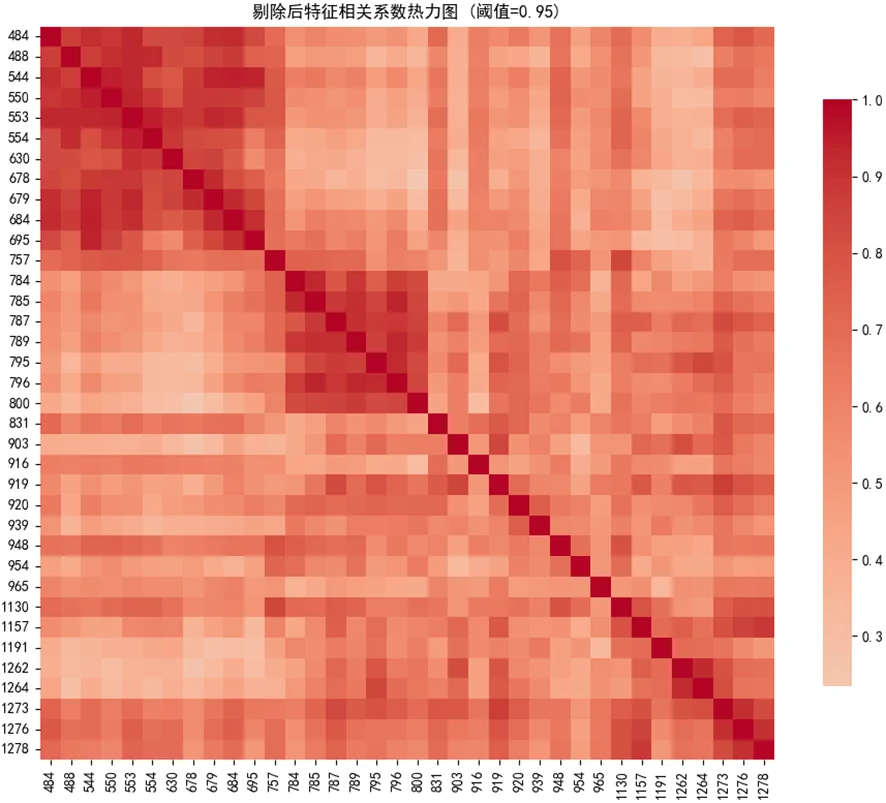
Results of highly correlated feature filtering.

Ground hyperspectral data are typically characterized by a large number of spectral bands, high spectral information density, and highly similar spectral responses across adjacent bands. These inherent properties readily give rise to massive redundant features and severe multicollinearity among variables. In this study, a filter-based feature selection method based on the Pearson correlation coefficient was applied to optimize spectral features. First, wavelengths with a correlation coefficient *r* above 0.5 were preliminarily retained. The linear correlation coefficients between each pair of candidate spectral bands were then calculated to identify and eliminate highly correlated features and uninformative spectral features with overlapping information (**Figure 7**). This procedure effectively reduces the dimensionality of hyperspectral features and mitigates the adverse impacts of multicollinearity among feature variables, while maximally preserving the valid spectral information contained in the original hyperspectral dataset.

#### 3.2.2 Feature selection

Five feature selection algorithms were employed to further refine the candidate bands pre-screened by the Pearson correlation coefficient method. The sensitive bands selected for winter wheat nitrogen inversion exhibited both consistencies and discrepancies across algorithms. As summarized in **Table 1**, bands at 544 nm, 554 nm, 678–679 nm, 757 nm, 916 nm, 939 nm, 1130 nm, 1191 nm, and 1262 nm were consistently selected by multiple algorithms. This indicates that these bands are sensitive to variations in winter wheat nitrogen status and represent critical candidate variables for characterizing plant nitrogen nutritional status. Specifically, bands in the visible region (484–695 nm) primarily reflect leaf chlorophyll content and are closely associated with plant nitrogen accumulation levels. Bands in the near-infrared range (750–1280 nm) capture differences in leaf cell structure, water content, and biomass, enabling effective differentiation of samples across nitrogen application gradients. Marked differences were observed in the number of bands retained by each algorithm. CARS selected the largest number of bands with relatively broad spectral coverage, whereas UVE retained the fewest bands, demonstrating a high degree of feature compression. The number of optimal bands selected by GA, LASSO, and VIP fell at an intermediate level. These divergences in selection outcomes stem from the distinct variable discrimination principles of each algorithm. CARS prioritizes the adaptive retention of bands carrying effective information; GA identifies optimal band combinations via evolutionary genetic optimization; LASSO achieves feature dimensionality reduction through coefficient shrinkage; and both VIP and UVE eliminate uninformative variables based on projection contribution rates. Overall, the optimally selected bands were concentrated in the typical sensitive visible and near-infrared spectral regions, which aligns with the established mechanism of crop nitrogen spectral response. These refined features provide a robust variable foundation for the subsequent development of quantitative winter wheat nitrogen inversion models.

**Table 1:** Feature information table.

Selection algorithm	Selected wavelengths/nm
CARS	484、544、554、630、678、679、757、785、789、796、916、1191、1262、1273、1278
GA	544、554、679、684、695、757、939、1130、1262
LASSO	550、678、695、757、903、916、939、954、965、1130、1191、1262、1278
VIP	757、831、903、916、920、939、954、965、1130、1191、1262、1273、1276、1278
UVE	544、679、757、796、916、939、1130、1157

### 3.3 Comparison of model performance

In this study, four machine learning algorithms and their stacking ensemble learning models were established for nitrogen content inversion based on seven datasets, namely SG, FD, FD-UVE, FD-VIP, FD-GA, FD-CARS and FD-LASSO. The corresponding results are presented in **Table 2**.

**Table 2:** Model accuracy evaluation under different datasets.

	SG		FD		FD-UVE		FD-VIP		FD-GA		FD-CARS		FD-LASSO	
	*R* ^2^	RMSE	*R* ^2^	RMSE	*R* ^2^	RMSE	*R* ^2^	RMSE	*R* ^2^	RMSE	*R* ^2^	RMSE	*R* ^2^	RMSE
RF	0.672	0.456%	0.766	0.386%	0.708	0.431%	0.777	0.376%	0.729	0.415%	0.763	0.388%	0.667	0.460%
SVR	0.670	0.458%	0.712	0.428%	0.715	0.425%	0.659	0.465%	0.737	0.408%	0.759	0.391%	0.738	0.408%
XGBoost	0.666	0.460%	0.748	0.400%	0.673	0.455%	0.808	0.349%	0.730	0.414%	0.776	0.377%	0.725	0.418%
GPR	0.572	0.521%	0.712	0.428%	0.696	0.439%	0.741	0.406%	0.712	0.428%	0.806	0.351%	0.871	0.286%
Stacking	0.727	0.416%	0.761	0.389%	0.736	0.410%	0.746	0.402%	0.771	0.381%	0.822	0.336%	0.875	0.281%

#### 3.3.1 Modeling differences between SG and FD spectra

**Figure 8:**
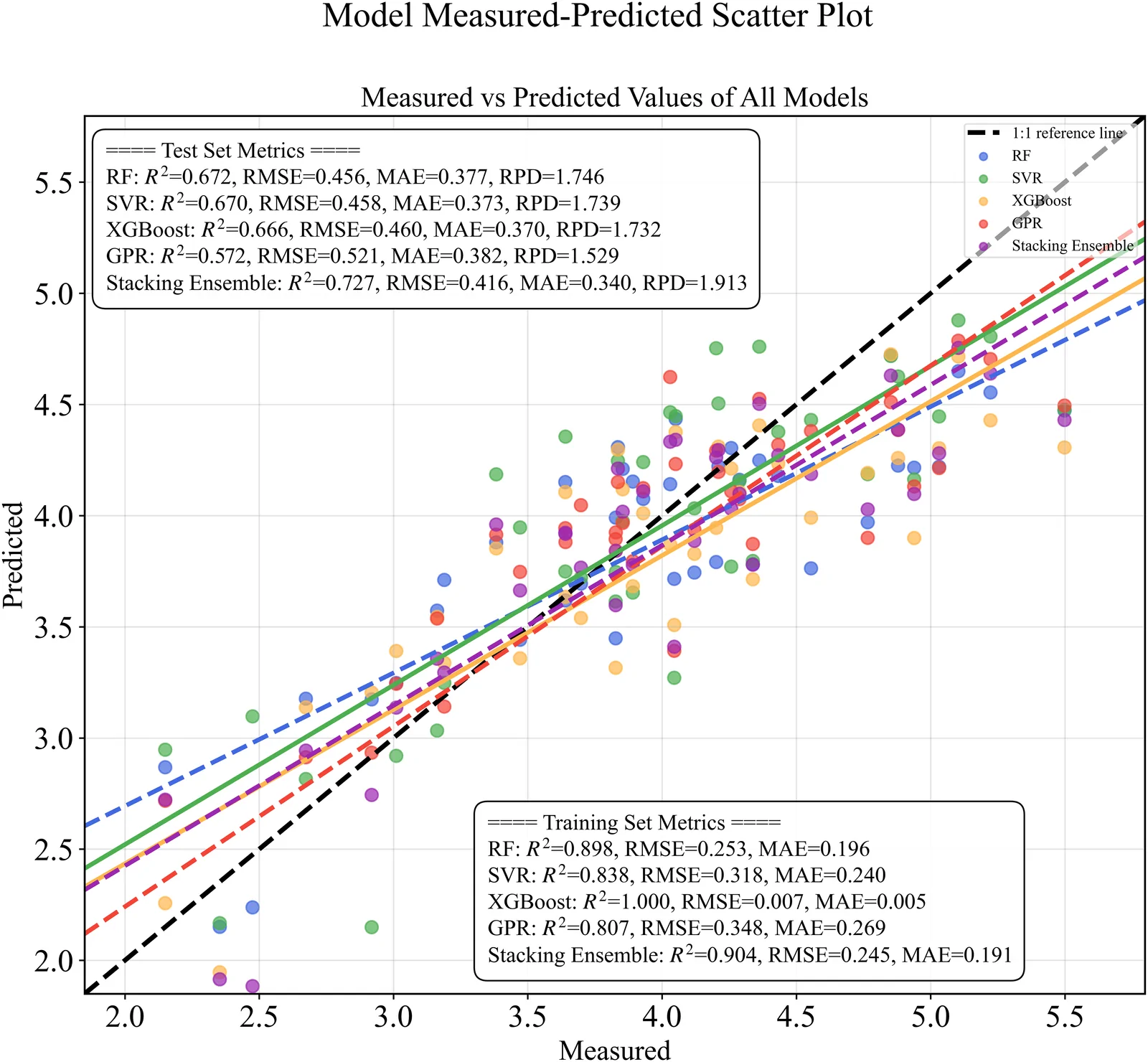
Prediction scatter plots for stacking ensemble model with SG smoothed spectra.

When only SG smoothed spectra were used as input, all models exhibited a pronounced performance gap between the training and test sets (**Figure 8**). XGBoost achieved a training-set *R*² close to 1.000, but its test-set *R*² dropped to only 0.666. This pattern suggests that XGBoost is relatively sensitive to high-dimensional noise and collinear features. GPR recorded the lowest test-set *R*² (0.572) among all models at this preprocessing stage. The stacking ensemble model yielded the best overall performance, with a test-set *R*² of 0.727 and an RMSE of 0.416%. This indicates that complementary error mitigation across multiple models helps alleviate overfitting. Nevertheless, raw high-dimensional spectral features still impose limitations on the generalization performance of the models.

**Figure 9:**
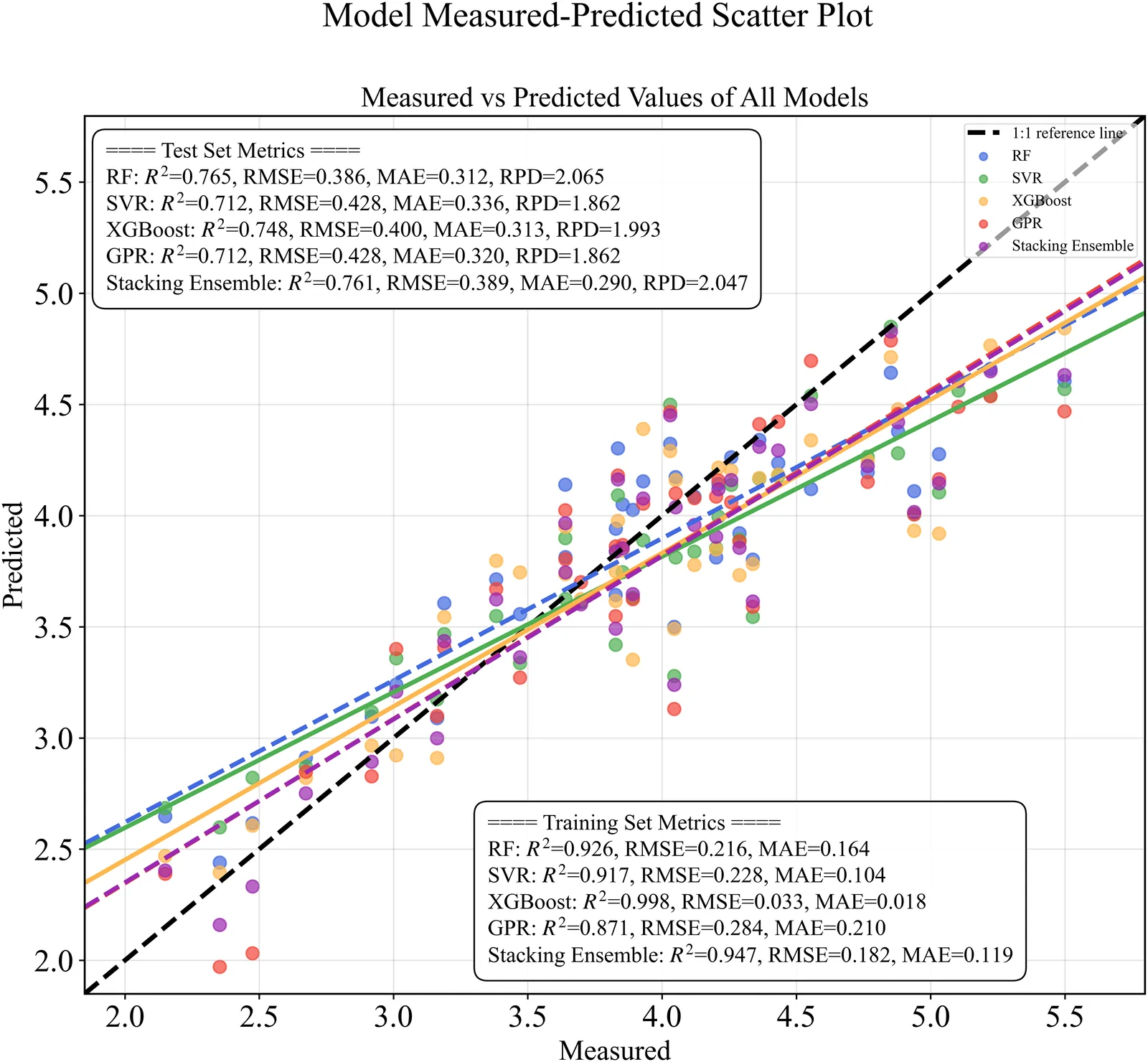
Prediction scatter plots for stacking ensemble model with FD spectra.

After FD transformation, the test-set *R*² of all models generally increased to 0.71–0.76 (**Figure 9**). RF achieved the highest test accuracy, with an *R*² of 0.766 and RMSE of 0.386%. The stacking ensemble model yielded comparable performance, with an *R*² of 0.761 and an RMSE of 0.389%. GPR exhibited the most pronounced performance improvement among all models. Although XGBoost still showed moderate overfitting, the performance gap between the training and test sets was substantially narrowed compared with the SG-only preprocessing stage. These results indicate that FD transformation enhances local spectral variation signals associated with nitrogen content. Nevertheless, spectral preprocessing alone is insufficient to fully mitigate the negative impacts of high-dimensional feature redundancy.

#### 3.3.2 Model adaptability under different feature subsets

**Figure 10:**
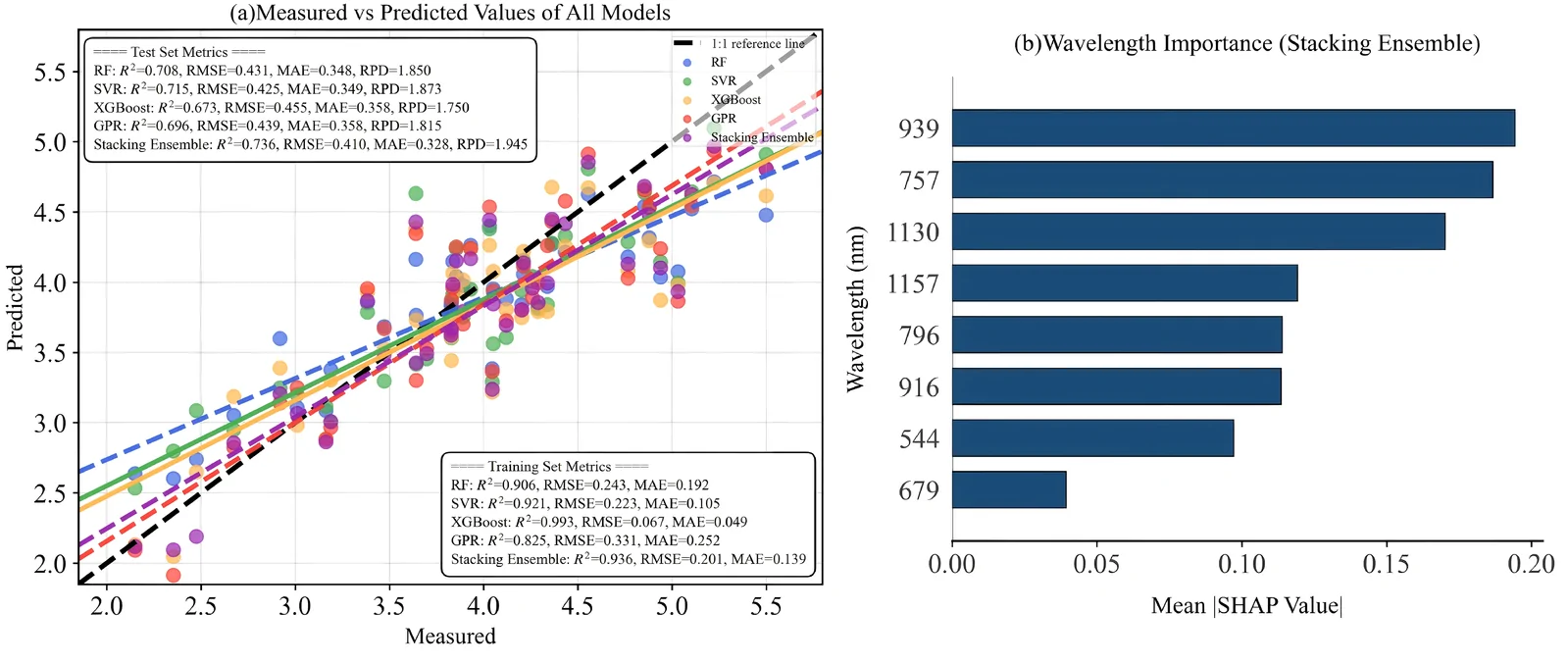
**(a)** Measured_versus_predicted scatter plot for all models using UVE_selected feature subsets; **(b)** Wavelength importance derived from SHAP analysis for the stacking ensemble model.

**Figure 11:**
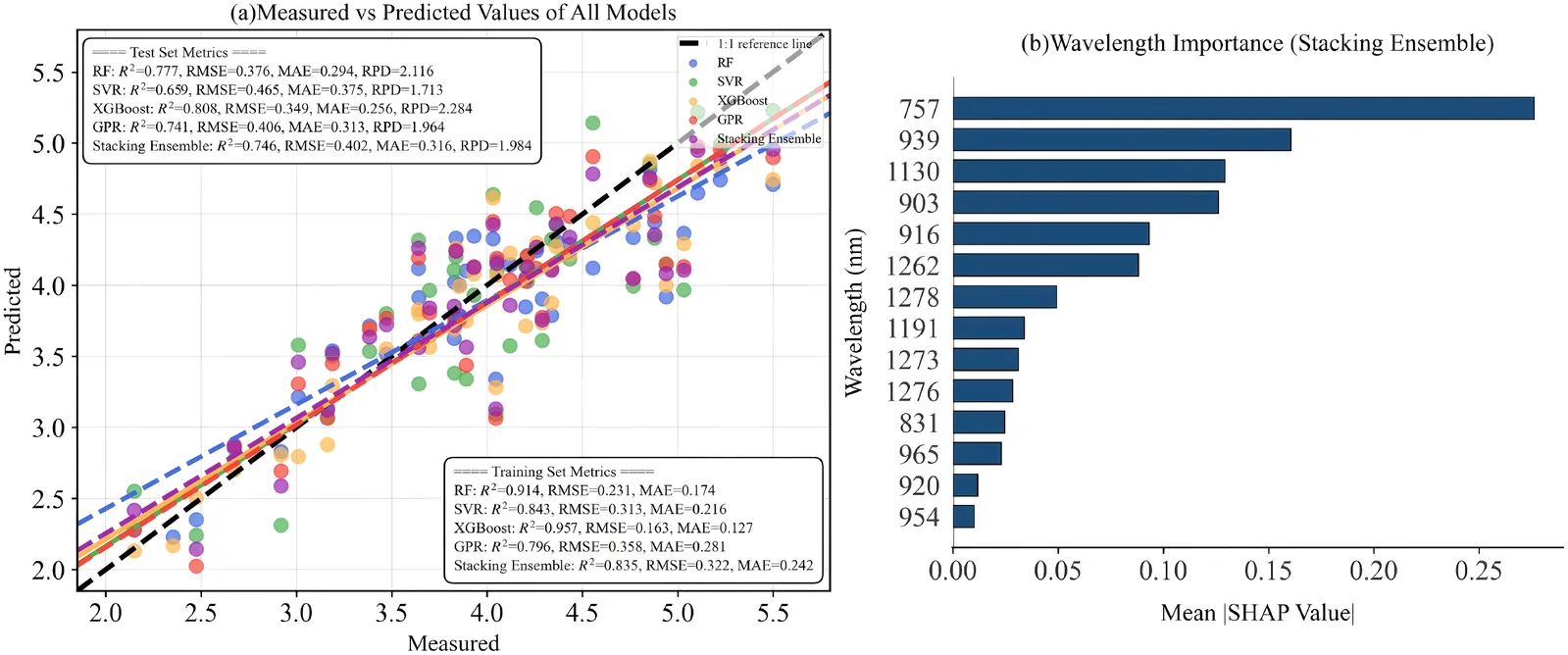
**(a)** Measured_versus_predicted scatter plot for all models using VIP_selected feature subsets; **(b)** Wavelength importance derived from SHAP analysis for the stacking ensemble model.

**Figure 12:**
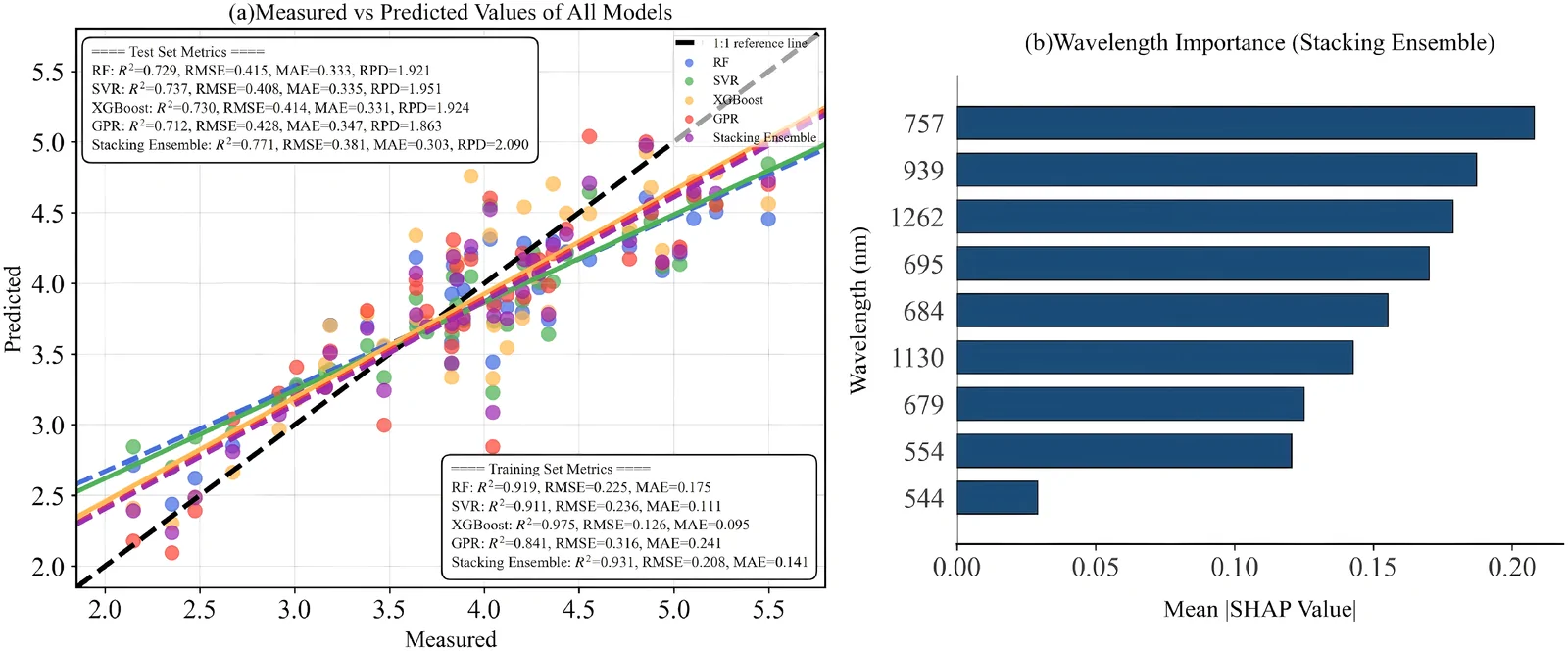
**(a)** Measured_versus_predicted scatter plot for all models using GA_selected feature subsets; **(b)** Wavelength importance derived from SHAP analysis for the stacking ensemble model.

**Figure 13:**
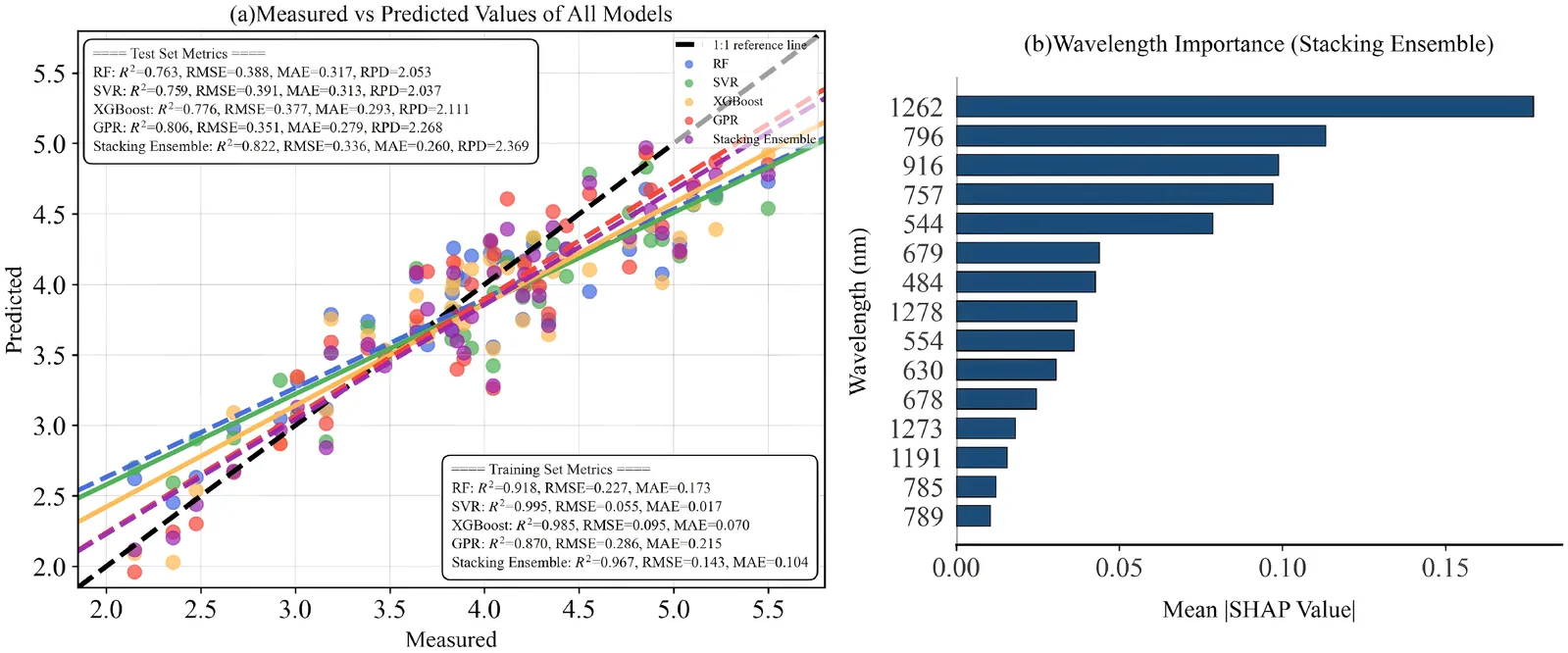
**(a)** Measured_versus_predicted scatter plot for all models using CARS_selected feature subsets; **(b)** Wavelength importance derived from SHAP analysis for the stacking ensemble model.

**Figure 14:**
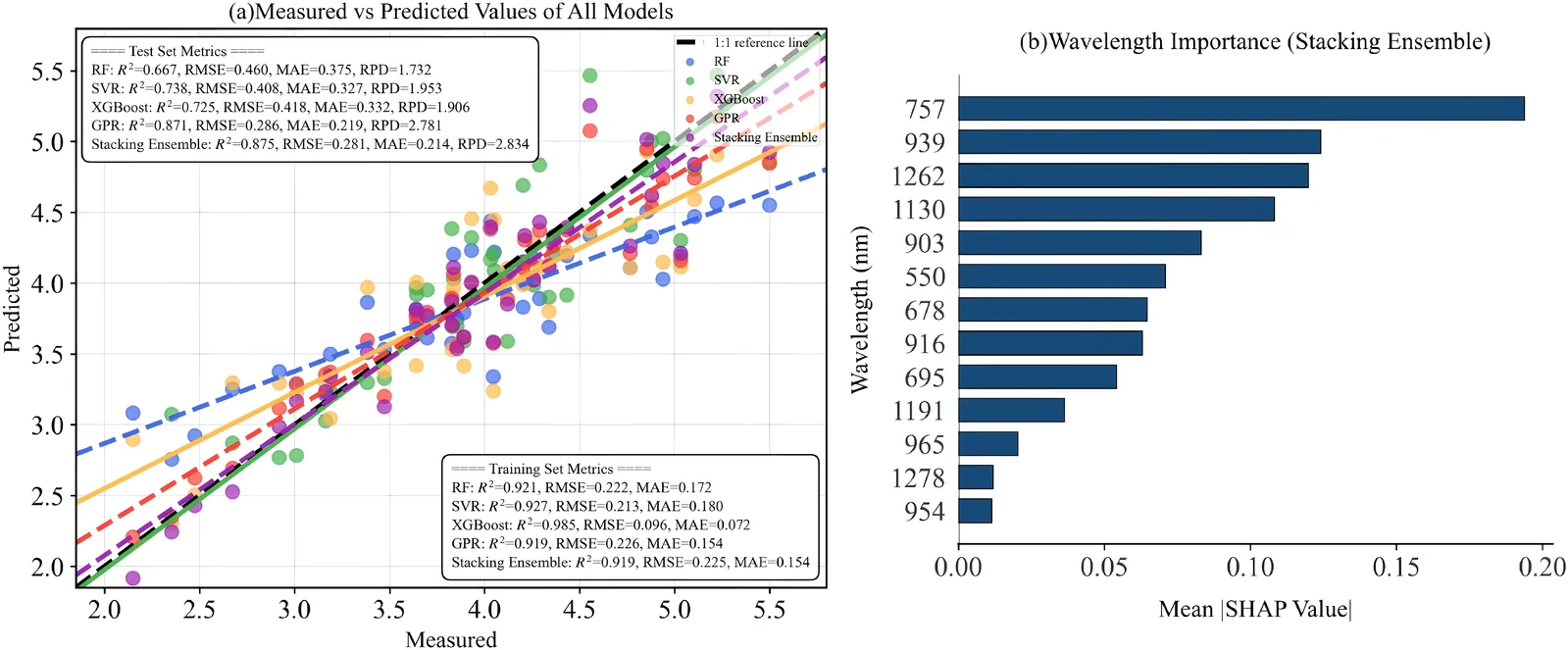
**(a)** Measured_versus_predicted scatter plot for all models using LASSO_selected feature subsets; **(b)** Wavelength importance derived from SHAP analysis for the stacking ensemble model.

(1)After feature selection via UVE, the stacking ensemble model achieved the best performance on this feature subset, with a test-set *R*² of 0.736, RMSE of 0.410%, MAE of 0.328%, and RPD of 1.945 (**Figure 10a**).SHAP-based interpretability analysis, presented in **Figure 10b**, reveals a distinct hierarchical pattern in the contribution of each wavelength to model inversion decisions. Bands at 939 nm, 757 nm, and 1130 nm were identified as core contributing wavelengths, collectively accounting for 53.15% of the total contribution. Bands at 1157 nm, 916 nm, and 796 nm served as secondary supporting variables, while 544 nm and 679 nm played only auxiliary supplementary roles. Overall, near-infrared bands contributed 86.67% of the total feature importance, acting as the primary information carrier for nitrogen concentration inversion. For individual base learners, SVR and RF yielded test-set *R*² values of 0.715 and 0.708, respectively. GPR delivered a reduced *R*² of 0.696. XGBoost still exhibited pronounced overfitting, with a training-set *R*² of 0.993 but a test-set *R*² of only 0.673.

(2)The VIP feature subset exhibited superior compatibility with the XGBoost model, which achieved a test-set *R*² of 0.808, RMSE of 0.349%, MAE of 0.256%, and RPD of 2.284 (**Figure 11a**), representing a marked improvement over XGBoost performance on the full FD spectrum. RF maintained stable performance (*R*² = 0.777, RMSE = 0.376%, MAE = 0.294%, RPD = 2.116), while the stacking ensemble model and GPR yielded *R*² values of 0.746 and 0.741, respectively. SVR showed a reduced *R*² of 0.659. SHAP-based interpretability analysis, presented in **Figure 11b**, demonstrates that the contribution of individual wavelengths to model inversion decisions follows a distinct long-tailed distribution. The 757 nm red edge band made the largest single contribution, accounting for 25.16% of total feature importance. Together with bands at 939 nm, 903 nm, and 1130 nm, it constituted the core dominant wavelengths, with a cumulative contribution of 64.05%. The remaining bands formed hierarchical tiers in descending order of contribution strength: key supporting bands, secondary auxiliary bands, and weak supplementary bands. All selected bands were concentrated in the red edge and near-infrared spectral regions. Visible bands with negligible contribution were effectively filtered out by the VIP feature selection procedure.

(3)After feature selection via GA, the stacking ensemble model achieved the best performance in this group, with a test-set *R*² of 0.771, RMSE of 0.381%, MAE of 0.303%, and RPD of 2.090 (**Figure 12a**). SVR and RF exhibited comparable performance, with test-set *R*² values of 0.737 and 0.729, respectively. XGBoost still exhibited the pattern of high fitting on the training set but limited performance improvement on the test set, with a training-set *R*² of 0.975 and a test-set *R*² of 0.730. GPR yielded a test-set *R*² of 0.712. SHAP-based interpretability analysis, presented in **Figure 12b**, reveals that the contribution of individual wavelengths to model inversion decisions follows a gradient differentiation pattern. Bands at 757 nm, 939 nm, 1262 nm, and 695 nm were identified as core dominant wavelengths, accounting for a cumulative contribution of 56.50%. Bands at 684 nm, 1130 nm, 679 nm, and 554 nm constituted important supports for inversion accuracy, while the 544 nm band played only a weak marginal auxiliary role. GA selection retained multiple characteristic wavelengths near the red light absorption peak. The combined contribution of visible and red edge bands reached 61.38%.

(4)The CARS feature subset yielded the most balanced performance across all models (**Figure 13a**). The stacking ensemble model achieved a test-set *R*² of 0.822, RMSE of 0.336%, MAE of 0.206%, and RPD of 2.369. GPR obtained a test-set *R*² of 0.806, RMSE of 0.351%, MAE of 0.279%, and RPD of 2.268, representing a marked improvement compared with the full FD spectrum. XGBoost and SVR recorded test-set *R*² values of 0.776 and 0.759, respectively, with a narrowed performance gap between the training and test sets. RF maintained stable performance (*R*² = 0.763). SHAP-based interpretability analysis, presented in **Figure 13b**, reveals that the contribution of individual wavelengths to model inversion decisions follows a long-tailed distribution pattern characterized by core dominant bands and multi-level supporting tiers. The 1262 nm mid-wave near-infrared band made the highest single contribution, accounting for 21.55% of total feature importance. Together with bands at 796 nm, 916 nm, and 757 nm, it constituted the core dominant wavelengths with a cumulative contribution of 57.70%. The remaining bands formed hierarchical tiers in descending order of contribution strength: key supporting bands, secondary auxiliary bands, and weak supplementary bands. Notably, CARS selection incorporated blue-sensitive bands for the first time, fully covering the complete spectral sensitive range from visible light, red edge, short-wave near-infrared to mid-wave near-infrared. This achieves comprehensive information coverage spanning from the physiological response of leaf pigments to the biochemical absorption of nitrogen-containing molecules.

(5)After feature selection via LASSO, the resulting feature subset delivered the highest test-set inversion accuracy among all feature selection schemes in this study (**Figure 14a**). GPR achieved a test-set *R*² of 0.871, RMSE of 0.286%, MAE of 0.219%, and RPD of 2.781. The stacking ensemble model further improved the inversion performance, with an *R*² of 0.875, RMSE of 0.281%, MAE of 0.214%, and RPD of 2.834.SVR maintained moderate robustness with an *R*² of 0.738, while overfitting in XGBoost was alleviated (*R*² = 0.725). Notably, RF exhibited a reduced *R*² of 0.667. This indicates that the excessively sparse feature subset diminished the variable diversity that underpins the predictive performance of RF. This finding demonstrates that the optimal feature selection algorithm does not necessarily deliver consistent superior performance across all model architectures. SHAP-based interpretability analysis, presented in **Figure 14b**, reveals that the contribution of individual wavelengths to model inversion decisions follows a balanced distribution pattern characterized by core leading bands and multi-level supporting tiers. The 757 nm red edge band made the largest single contribution, accounting for 19.92% of total feature importance. Together with the 1262 nm, 939 nm, and 1130 nm bands, it formed the core dominant wavelengths, with a cumulative contribution of 57.76%. The selected bands fully covered four major nitrogen-sensitive spectral regions: visible light, red edge, short-wave near-infrared, and mid-wave near-infrared. The contribution distribution across each spectral region was more balanced compared with other feature selection schemes.

#### 3.3.3 Cross-growth stage inversion based on the optimal model

**Figure 15:**
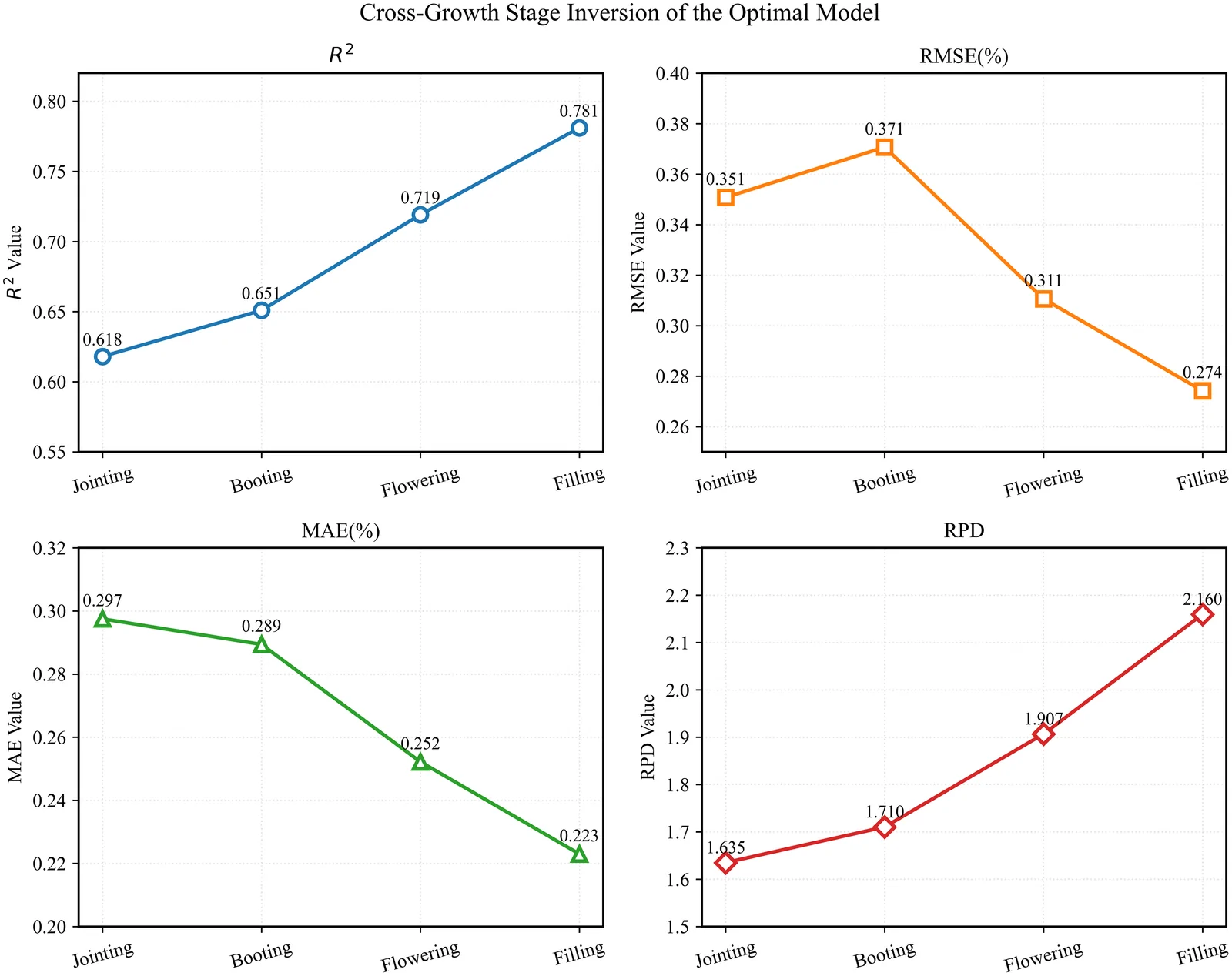
Cross-growth stage inversion of the LASSO-Stacking model.

In this study, the LASSO-Stacking model was selected for cross-growth stage inversion. One growth stage out of the four was taken as the test set in turn, and the remaining three stages were used as the training set for iterative modeling and training. **Figure 15** illustrates the cross-growth stage inversion results of the LASSO-Stacking model, among which the highest accuracy was achieved at the filling stage with an *R*² of 0.781.

## 4 Discussion

This study developed a three-tier framework—spectral preprocessing, feature selection, and model architecture—for hyperspectral retrieval of leaf nitrogen in winter wheat, and employed SHAP to reveal band-wise contribution mechanisms, ultimately achieving a test-set *R*² of 0.875. Unlike previous studies that optimize a single stage, our work clarifies the selective enhancement mechanism of the FD transform on the N signal, quantifies the compatibility between five feature-selection algorithms and four models, and uses SHAP to extract physiologically meaningful band-contribution hierarchies from otherwise black-box models. The synergy across the three tiers and the SHAP interpretability are discussed below.

### 4.1 Signal-level regulation by FD preprocessing

In the raw SG spectra, the “three-high/three-low” correlation pattern with N concentration showed high correlations in the shortwave-infrared (SWIR) that essentially arise from indirect proxy signals of the N–water–dry-matter coupling chain, which are strongly confounded and poorly transferable. The FD transform reshaped the spectral response through three effects. First, it removed low-frequency interference such as baseline drift, exposing the fine absorption features masked in the 400–1100 nm visible–red-edge region. Second, it amplified slope changes at the red edge and absorption shoulders, producing the strongest isolated peak across the full spectrum at the leading edge of the 1200–1300 nm water-absorption band (r = 0.65–0.70), which became the most N-sensitive waveband; this is consistent with the high sensitivity of the red-edge region to canopy biochemical and structural traits (Sun et al., 2020). Third, it removed the slowly varying coupled signals in the SWIR, causing correlations to decay rapidly beyond 1300 nm. It should be noted that the N-related protein N–H and C–N overtone/combination bands are physically centered near ~1500 nm and 2050–2180 nm; the observed near-infrared (NIR) response therefore more likely reflects a composite N–protein–structure signal, whose separation into direct and indirect components still requires radiative transfer modeling. Overall, FD achieves a selective regulation that enhances the direct physiological response while stripping indirect confounding signals. To suppress the high-frequency noise introduced by differentiation, an “SG smoothing + FD” strategy was adopted to balance enhancement and denoising.

### 4.2 Structural differences among feature subsets and their compatibility with models

The five algorithms produced markedly different feature structures owing to their distinct selection mechanisms: CARS retained variables through Monte-Carlo competition and adaptive reweighting, yielding the broadest, continuous and balanced subset (15 bands); GA searched optimal combinations by evolutionary optimization, yielding a pigment-oriented subset (9 bands, 61.38% in the visible–red-edge); LASSO shrank coefficients via L1 regularization, yielding a sparse, high-purity subset (13 bands); VIP selected bands by PLS projection importance, yielding an NIR-dominated subset (14 bands, excluding the visible); and UVE eliminated variables by regression-coefficient stability, yielding the most parsimonious subset (8 bands). The degree of match between feature structure and a model’s inductive bias governed retrieval accuracy, as verified by SHAP. GPR was highly compatible with LASSO (*R*² = 0.871), because Gaussian-process kernels measure similarity more accurately on low-redundancy, high-purity features; SHAP showed balanced, non-redundant contributions across the four band intervals of the LASSO subset. XGBoost matched VIP best (*R*² = 0.808), consistent with the alignment between VIP projection importance and the split-gain logic of tree models; here SHAP indicated that the single band at 757 nm (the red-edge–NIR transition) contributed 25.16% and dominated the decision. CARS showed the strongest cross-model transferability, owing to its broad coverage and moderate redundancy, with 1262 nm contributing most (21.55%) while spanning the full visible-to-NIR range. In contrast, RF declined on the LASSO subset (*R*² from 0.766 to 0.667), confirming its dependence on feature diversity—excessive sparsity raises inter-tree correlation and degrades the ensemble. Unlike previous crop-trait studies that mainly compared the overall accuracy of several algorithms under a single model (Guo et al., 2023), our unified framework systematically characterizes the “feature-structure–model-bias” compatibility.

### 4.3 Three-tier synergy and SHAP interpretability

The optimal combination, FD-LASSO-Stacking (*R*² = 0.875, RMSE = 0.281%, RPD = 2.834), resulted from the synergy of the three tiers, forming a clear accuracy-improvement chain: SG-full-spectrum Stacking (*R*² = 0.727) → FD-full-spectrum Stacking (*R*² = 0.761, + 4.7%) → FD-LASSO Stacking (*R*² = 0.875, + 15.0%). The feature-selection stage contributed the most, indicating that in hyperspectral retrieval “selecting the right features” is more critical than “selecting the right model.” For winter wheat with the same FD preprocessing, Chen et al. (2025) reported a best model of FD combined with Elastic Net (*R*² = 0.78, RMSE = 0.28%, RPD = 2.15); at a comparable RMSE, the present framework raised *R*² and RPD to 0.875 and 2.834, respectively, demonstrating the gain of three-tier synergy over single-stage optimization. When the FD-LASSO-Stacking model was adopted for cross-growth-stage inversion, the best performance was obtained at the grain-filling stage with an *R*^2^ of 0.78. This result verifies the applicability and moderate generalization ability of the model to a certain extent.

Error complementarity among base learners underpins the stacking gain. RF, SVR, XGBoost, and GPR have distinct inductive biases and therefore different error patterns; when these errors are weakly correlated, the meta-learner offsets systematic bias and smooths random fluctuations through weighting. SHAP further showed that, under the high-purity LASSO input, the error boundaries of the base learners became clearer and their complementary fusion more efficient; thus, although GPR alone already reached *R*² = 0.871, stacking still yielded a marginal improvement.

SHAP translated model decisions into physiologically meaningful band-contribution hierarchies. First, 757 nm (the red-edge–NIR transition) was a core cross-algorithm band (25.16% in VIP, 19.92% in LASSO), corroborating the role of the red-edge inflection as a classical N indicator. Second, quantifying the four response windows revealed that the NIR structure–physiology region accounted for more than 60% of the contribution (up to 86.67% in the UVE subset), indicating a continued reliance on indirect coupled signals. Third, the selection logic of different algorithms corresponded to distinguishable physiological responses, providing a physiological—rather than purely statistical—basis for feature selection. Compared with recent crop-retrieval studies that achieve “interpretability” via attention mechanisms yet remain accuracy-oriented (Li et al., 2025b), our approach maps band contributions directly onto physiological response windows, strengthening the mechanistic credibility of the results.

### 4.4 Limitations and prospects

Three limitations remain. First, the spatiotemporal representativeness of the data is limited: although the single-site, three-cultivar design ensured a clear N gradient and an approximately normal distribution (Shapiro–Wilk, p = 0.1461), cross-regional generalization requires validation through multi-ecoregion trials. Second, upscaling needs re-evaluation: when extending from ground canopy spectra to airborne and spaceborne platforms, the noise sensitivity of FD and mixed-pixel effects may alter the optimal preprocessing strategy. Third, mechanistic decoupling should be deepened: the cell-structure, water, and dry-matter effects underlying the high NIR contribution still need to be separated with radiative transfer models such as PROSPECT. Future work could integrate radiative transfer mechanisms within the SHAP interpretability framework to build hybrid retrieval models that combine accuracy with physical meaning.

## 5 Conclusions

Based on field-measured canopy hyperspectral data and leaf nitrogen content of three winter wheat varieties under four nitrogen application gradients across the jointing to filling stages at the Jiaozuo experimental site, this study constructed a three-level progressive inversion framework integrating spectral preprocessing, feature screening, and stacked ensemble modeling. With SHAP interpretability analysis, the combined effects of two preprocessing methods, five feature screening algorithms, and five modeling schemes were systematically quantified, and the physiological basis underlying model decision-making was clarified. At the preprocessing level, FD achieves selective regulation of spectral signals by eliminating low-frequency noise, amplifying red-edge features, and decoupling mixed signals in the short-wave infrared band. It elevates the model test-set *R*² to 0.71–0.76 and effectively mitigates overfitting. At the feature screening level, feature selection yields markedly stronger optimization than model-structure tuning, with distinct adaptive rules: LASSO pairs well with GPR, VIP suits XGBoost, and CARS delivers the best generalization performance, whereas UVE performs poorly due to loss of valid spectral information. The optimized FD-LASSO-stacked ensemble model attained the best inversion performance (*R*² = 0.875, RMSE = 0.281%, RPD = 2.834), representing a 15.0% accuracy improvement over the full-spectrum model. Mechanistically, SHAP analysis identified 757 nm and 939 nm as the core response bands for winter wheat leaf nitrogen inversion, confirming the biological rationality of model decisions. Meanwhile, the stable bands retained by CARS provide a scientific reference for developing portable multispectral sensors. By enabling accurate, real-time monitoring of crop nitrogen status across the growing season, this framework provides critical technical support for implementing mass-balance-based nitrogen management in winter wheat, supporting precise fertilizer input, improved nitrogen use efficiency, and reduced agricultural non-point source pollution in intensive wheat production systems.

## Data Availability

The raw data supporting the conclusions of this article will be made available by the authors, without undue reservation.
